# Survey of subjective "God encounter experiences": Comparisons among naturally occurring experiences and those occasioned by the classic psychedelics psilocybin, LSD, ayahuasca, or DMT

**DOI:** 10.1371/journal.pone.0214377

**Published:** 2019-04-23

**Authors:** Roland R. Griffiths, Ethan S. Hurwitz, Alan K. Davis, Matthew W. Johnson, Robert Jesse

**Affiliations:** 1 Department of Psychiatry and Behavioral Sciences, Johns Hopkins University School of Medicine, Nathan Shock Drive, Baltimore, Maryland, United States of America; 2 Department of Neuroscience, Johns Hopkins University School of Medicine, Nathan Shock Drive, Baltimore, Maryland, United States of America; 3 Department of Psychology, University of California San Diego, Gilman Drive, San Diego, California, United States of America; 4 Council on Spiritual Practices, Occidental, California, United States of America; University of Auckland, NEW ZEALAND

## Abstract

Naturally occurring and psychedelic drug–occasioned experiences interpreted as personal encounters with God are well described but have not been systematically compared. In this study, five groups of individuals participated in an online survey with detailed questions characterizing the subjective phenomena, interpretation, and persisting changes attributed to their single most memorable God encounter experience (n = 809 Non-Drug, 1184 psilocybin, 1251 lysergic acid diethylamide (LSD), 435 ayahuasca, and 606 *N*,*N*-dimethyltryptamine (DMT)). Analyses of differences in experiences were adjusted statistically for demographic differences between groups. The Non-Drug Group was most likely to choose "God" as the best descriptor of that which was encountered while the psychedelic groups were most likely to choose "Ultimate Reality." Although there were some other differences between non-drug and the combined psychedelic group, as well as between the four psychedelic groups, the similarities among these groups were most striking. Most participants reported vivid memories of the encounter experience, which frequently involved communication with something having the attributes of being conscious, benevolent, intelligent, sacred, eternal, and all-knowing. The encounter experience fulfilled *a priori* criteria for being a complete mystical experience in approximately half of the participants. More than two-thirds of those who identified as atheist before the experience no longer identified as atheist afterwards. These experiences were rated as among the most personally meaningful and spiritually significant lifetime experiences, with moderate to strong persisting positive changes in life satisfaction, purpose, and meaning attributed to these experiences. Among the four groups of psychedelic users, the psilocybin and LSD groups were most similar and the ayahuasca group tended to have the highest rates of endorsing positive features and enduring consequences of the experience. Future exploration of predisposing factors and phenomenological and neural correlates of such experiences may provide new insights into religious and spiritual beliefs that have been integral to shaping human culture since time immemorial.

## Introduction

Salient experiences interpreted as personal encounters with God, gods, or emissaries of God, have been documented for millennia, have been integral to the development of religious and spiritual beliefs, and have had a major influence in shaping human culture [1–5]. Such experiences, which often occur unexpectedly and in absence of drugs or physical illness, may involve visions, voices, or what is felt to be a mental or extrasensory apprehension of that which is encountered. Descriptions of such experiences sometimes overlap with mystical-type experiences, which have also been well documented and have been a focus of substantial empirical research [3,6]. The majority of rigorous empirical studies of mystical experiences [7–11] have used the Hood M Scale, which is based on the conceptual model of mystical experience described by Stace [4] and emphasizes a sense of unity as a central defining characteristic of mystical experience. Stace [4], but not all scholars of religion [12], explicitly exclude vision and voice phenomena from the descriptive definition of mystical experience thus suggesting that some God encounter experiences may be more appropriately classified as religious but not mystical experiences.

God encounter and mystical experiences have also been described after ingestion of classic psychedelic drugs such as psilocybin, lysergic acid diethylamide (LSD), *N*,*N*-dimethyltryptamine (DMT), and mescaline, all of whose actions are mediated at the serotonin 2A receptor [13–15]. Historically, the use of psychedelic-containing plants and fungi in ceremonial and religious contexts dates back hundreds and likely thousands of years [16–19]. Indeed, the Aztecs called one or more species of psilocybin mushrooms Teonanácatl, which is translated as "flesh of the gods" or "God’s flesh" [17,18]. More recently, the classic psychedelics have sometimes been called "entheogens," a term derived from ancient Greek meaning "becoming God within" and used to refer to plants or drugs ingested in a religious context for spiritual purposes [20]. Contemporary use of the classic psychedelics in formal religious or spiritual contexts include the use of mescaline in the peyote cactus by Native American Indians [21,22], and the use of DMT in ayahuasca by several religious groups most prominently represented by the Santo Daime and União do Vegetal churches which originated in South America and have more recently been established throughout the world [23]. In addition, spiritual exploration is reported to be a primary motive for contemporary illicit use of classic psychedelics [24,25].

In addition to historical and contemporary reports of religious and spiritual use of psychedelics, a series of double-blind studies using the Hood M Scale, which was developed to measure naturally occurring mystical experiences, showed that the classic psychedelic compound psilocybin could reliably and dose-dependently occasion salient mystical experiences in healthy psychedelic-naïve participants, most of whom had no history of having had a spontaneously occurring mystical experience [26–28]. An extension of this research developed and validated the Mystical Experience Questionnaire (MEQ30) for measuring single psychedelic-occasioned mystical experiences [29,30]. Like the M Scale, the MEQ30 is based on the conceptual model of mystical experience described by Stace [4], which emphasizes a sense of unity and does not make reference to God. Although previous laboratory studies of psilocybin did not assess God encounter experiences per se, some participants in the laboratory studies spontaneously described such experiences [31].

Despite the compelling empirical similarity between naturally-occurring and psychedelic-occasioned mystical experiences, there has been debate among scholars of religion about whether or not mystical experiences occasioned by psychedelics can be considered to be "genuine" mystical or religious experiences. Although some have argued, largely on conceptual grounds, that drug-induced experiences are not religious experiences [32–34], others have argued for and cited indirect empirical support suggesting the equivalence of naturally occurring and psychedelic mystical experiences [35,36].

The present study was undertaken to advance our understanding of both naturally occurring and psychedelic-occasioned religious experiences that are interpreted as an encounter with God (e.g., the God of your understanding), Higher Power, Ultimate Reality, or an Aspect or Emissary of God (e.g., an angel). [*Nota bene*: To simplify the writing of the present report, the term "God encounter experience" will be used as a label to refer to all four descriptive variants of these experiences. We have chosen to capitalize the word "God" to be consistent with the survey instructions and question wording.] This study was an internet survey of a large international sample of individuals who reported having had such an experience. Detailed questions were asked to characterize participant demographics and the subjective phenomena, interpretation, and persisting changes attributed to their single most memorable God encounter experience. The data allowed comparison between those who did and did not ingest a psychedelic drug, comparison among four different types of classic psychedelic substances (psilocybin, LSD, ayahuasca, and DMT), and examination of whether such God encounter experiences fulfill criteria for being mystical experiences.

## Methods

### Participant recruitment

Participants were recruited primarily via internet advertisements, email invitations, and online social networks. Two different participant groups were recruited corresponding to two versions of the questionnaire. The purpose of both was stated as: "In this survey, we want to characterize various experiences of encounters with something that someone might call: God (e.g., the God of your understanding), Higher Power, Ultimate Reality, or an Aspect or Emissary of God (e.g., an angel)." However, one group (the Psychedelic Group) completed the questionnaire based on an experience of encountering something that occurred after taking a classic hallucinogen (e.g., psilocybin, LSD, ayahuasca, DMT, etc.). The other group (the Non-Drug Group) completed the questionnaire based on an experience that occurred in absence of taking a psychoactive drug. Internet and email advertisements provided a webpage link to the appropriate version of the questionnaire. Participants were informed that study participation was anonymous, they could choose to stop answering questions at any time, and if they did not complete the questionnaire their specific responses would not be used. The Institutional Review Board of the Johns Hopkins University School of Medicine approved all study procedures.

### Survey administration

The questionnaires were designed to take approximately 50 minutes to complete and participants were required to complete the survey in one sitting. The questionnaires were hosted on a widely used online survey administration website (www.qualtrics.com) with security and privacy features that make it suitable for anonymous survey data collection and storage. No compensation was provided for study completion.

### Inclusion criteria

Participants in the psychedelic version of the questionnaire were required to fulfill the following inclusion criteria: (1) Were at least 18 years old; (2) Read, write, and speak English fluently; (3) Had not completed the questionnaire previously; (4) Had a God encounter experiences (as described above) after taking a dose of a classic hallucinogen that had moderate to strong psychoactive effects. Participants in the non-drug version of the questionnaire were required to fulfill inclusion criteria 1, 2, and 3 as well as the additional criterion that they had had a God encounter experience (as described above) but that they had never had such an experience after having taken a psychoactive drug. This final exclusion criterion assured that responses from non-drug respondents were not confounded by having had a drug-occasioned God encounter experience.

Participants who met the inclusion criteria were directed to the remaining items in the questionnaire. In completing the questionnaire, participants were instructed to answer the items in reference to their single most memorable experience.

### Survey description

Details of questionnaire items are provided in the Results. Briefly, participants answered basic demographic questions, wrote a brief textual description of their encounter experience, and answered a series of questions about the details of their experience such as the style of communication (e.g. visual, auditory), their interpretation of qualities of that which was encountered (e.g., benevolent, intelligent, sacred), and persisting changes attributed to the experience. Within the survey questionnaire, participants also completed the Mystical Experience Questionnaire (MEQ30) [29,30] with the instructions to answer questions according to their feelings, thoughts, and experiences at the time of the encounter. Complete mystical experience was defined *a priori* as having scores 60% or above on all four MEQ30 subscales [30]. Participants in the psychedelic version of the study indicated which of several classic hallucinogens they believe they had taken: psilocybin mushrooms, psilocybin, LSD (acid), ayahuasca, DMT (other than ayahuasca), mescaline, peyote cactus, or other.

### Statistical analyses

Data analysis for psychedelic drug users was restricted to those who indicated they had taken one of the four major categories of psychedelic drugs: psilocybin, LSD, ayahuasca, and DMT (other than ayahuasca).

Demographic data: For demographic comparisons between the Non-Drug Group and the Psychedelic Group, dichotomous variables were analyzed with Chi-square and continuous variables were analyzed with ANOVA. For demographic comparisons among the non-drug, psilocybin, LSD, ayahuasca, and DMT groups, dichotomous variables were analyzed with a general linear model with a logit link and continuous variables were analyzed with ANOVA. For pairwise comparisons among groups, Bonferroni corrections were used to control for Type I error rate.

Comparison of ratings of experience between the Non-Drug Group and the Psychedelic Group: Dichotomous data for: 1. endorsement and non-endorsement of questionnaire items, and 2. complete and incomplete mystical experiences were coded as 1 and 0, respectively, and were analyzed using a general linear model in SPSS 24.0.0.0, with a logit link and Type III Sums of Squares. The following dichotomous covariates, which differed between the Non-Drug and Psychedelic groups (see Results), were included in the model: age at time of study participation (>32 years), age at time of experience (>23 years), sex, White race, college graduate, U.S. resident, income (>$50K/year), ever married. Continuous data were analyzed using ANOVA with the same covariates and Type III Sums of Squares.

Comparison of ratings of experience among the Non-Drug, Psilocybin, LSD, Ayahuasca, and DMT groups: Dichotomous data were coded as described above and were analyzed using a general linear model in SPSS, with a logit link and Type III Sums of Squares, including the eight covariates described above. Continuous data were analyzed using ANOVA with the same covariates and Type III Sums of Squares. For both sets of analyses, pairwise comparisons among the groups were adjusted using the Bonferroni method to control for Type I error rate.

Results for all of the analyses described above, including those which also used a Bonferroni correction, were considered significant when p≤0.001. These conservative statistical criteria were used in order to focus on robust differences between groups.

Religious orientation data: Comparisons among Non-Drug and Psychedelic groups for three religious orientation categories (atheist, monotheist, and other) were analyzed using Chi square tests. Pairwise comparisons among groups were conducted using z-tests for independent proportions. For comparison of changes in religious orientation before and after the encounter experience, z-tests of dependent proportions were used within each group. Bonferroni corrections were used to control for Type I error rate. Results were considered significant when the adjusted p≤0.05.

In the Results section, tables with dichotomous measures present percentage of participants in the group who endorsed the item or showed the effect; tables of continuous measures show means and standard deviations of the group. For completeness, supplemental tables show estimated means and standard errors of the estimate from the statistical analyses. For the dichotomous measures, the difference between the group percentage data (expressed as a proportion) and the estimated means were relatively small, with the mean difference across measures of 0.01 (range 0.00–0.08).

## Results

### Survey completion

During recruitment (12/03/2014–08/01/2016), 12,725 individuals began the survey. Of these, 1,702 were excluded because they did not meet the inclusion criteria, and 5,165 were excluded because they did not complete the questionnaire, with 82% and 93% of these failing to complete 25% and 50%, respectively, of the questionnaire items. Additionally, 401 were excluded because they indicated taking multiple substances, 602 because they reported taking a substance other than psilocybin, LSD, ayahuasca, or DMT, 271 because they answered the survey based on multiple rather than a single encounter experience, 104 because their responses raised concerns about the validity of their data overall, 34 because they indicated at the end of the survey that they did not want their responses included in the analyses, and 161 because of nonsystematic coding errors. Thus 4,285 individuals provided useable data. The median time to complete the questionnaire was 50 minutes. A written response in the open-ended comment section at the end of the questionnaire was provided by 67% of participants.

### Participant characteristics

Tables 1 and 2 present participants’ reported characteristics for the different participant subgroups. Participants were, on average 38.3 years of age at the time of the survey. Sixty-nine percent were male, 88% were White, and 48% had a college or graduate degree. Participants were, on average 27.2 years of age at the time of their experience, which occurred on average 11.0 years before completing the study.

**Table 1:** Participant characteristics in the Non-Drug Group and combined Psychedelic Group^1^.

Measure	Non-Drug Group (N = 809)	Psychedelic Group (N = 3476)	*P* value^2^
Age at time of study participation in years (mean, SD)	56.2 (13.7)	34.1 (12.8)	p≤0.001
Age at time of encounter experience in years (mean, SD)	35.7 (15.0)	25.3 (9.1)	p≤0.001
Years since the experience (mean, SD)	20.5 (15.4)	8.8 (10.6)	p≤0.001
Sex (% male)	27%	79%	p≤0.001
Race (%)^3^			p≤0.001
White	93%	86%	
Black/African American	1%	1%	
Asian	2%	3%	
Native Hawaiian or Pacific Islander	0%	0%	
Native American	1%	1%	
Mixed Race	3%	9%	
Ethnicity (% Hispanic)	4%	9%	p≤0.001
Education (%)^4^			p≤0.001
No high school diploma or equivalent (GED)	1%	3%	
High school diploma or equivalent (GED)	2%	13%	
Some college or vocational training	23%	42%	
Bachelor’s degree	30%	25%	
Master’s degree	27%	12%	
Advanced professional degree	16%	5%	
Annual household income (%)^5^			p≤0.001
Under $25,000	11%	36%	
$25,000—$49,999	22%	27%	
$50,000—$74,999	17%	13%	
$75,000—$99,999	13%	8%	
$100,000—$150,000	21%	10%	
$150,000 +	15%	6%	
Ever Married (%)	76%	46%	p≤0.001
Country of residence (%)^6^			p≤0.001
United States	75%	59%	
Canada, Europe, Australia	21%	31%	
Other (%)	4%	10%	

^1^ Subject characteristics at time of survey completion unless otherwise specified.

^2^ Dichotomous demographic variables were analyzed with Chi-square to compare between Non-Drug Group and Psychedelic Group. Continuous demographic variables were analyzed with ANOVA. Results were considered significant when p≤0.001.

^3^ Proportion White race compared between Non-Drug Group and Psychedelic Group.

^4^ Proportion having bachelor’s degree or higher compared between Non-Drug Group and Psychedelic Group.

^5^ Proportion having income less than $50,000 compared between Non-Drug Group and Psychedelic Group

^6^ Proportion with United States country of residence compared between Non-Drug Group and Psychedelic Group.

**Table 2:** Participant characteristics for Non-Drug, psilocybin, LSD, ayahuasca, and DMT groups^1^^,^^2^^,^^3^.

Measure	Non-Drug Group (N = 809)	Psilocybin Group (N = 1184)	LSD Group (N = 1251)	Ayahuasca Group (N = 435)	DMT Group (N = 606)
Age at time of study participation in years (mean, SD)	56.2 (13.7)	**33.0 (11.4)**^c^	**35.0 (14.8)**^b^	**40.5 (12.2)**^a^	**30.0 (8.3)**^d^
Age at time of encounter experience in years (mean, SD)	35.7 (15.0)	**25.1 (9.0)**^b^	**22.1 (5.9)**^c^	35.1 (11.3)^a^	**25.3 (7.8)**^b^
Years since the experience (mean, SD)	20.5 (15.4)	**7.9 (8.1)**^b^	**12.9 (14.5)**^a^	**5.3 (4.1)**^c^	**4.7 (3.3)**^c^
Sex (% male)	27%	**81%**^b^	**80%**^b^	**66%**^a^	**82%**^b^
Race (% White)	93%	**85%**^a^	**87%**^a^	**86%**^a^	87%^a^
Ethnicity (% Hispanic)	4%	**10%**^a^	**8%**^a^	**13%**^a^	8%^a^
Education (% Bachelor’s college degree or higher)	74%	**42%**^a^	**39%**^a^	**62%**^b^	**33%**^a^
Annual household income (% <$50,000)	33%	**65%**^a^	**62%**^a^	**56%**^a^	**67%**^a^
Ever Married (%)	76%	**45%**^b^	**45%**^b^	**61%**^a^	**39%**^b^
Country of residence (% United States resident)	75%	**60%**^a^	**63%**^a^	**44%**^b^	**63%**^a^

^1^ Participant characteristics at time of survey completion unless otherwise specified.

^2^ Within a row, bold font indicates significant difference from the Non-Drug Group; for the drug groups, values not sharing a common letter are significantly different.

^3^ Dichotomous demographic variables were analyzed with a general linear model with a logit link. Continuous demographic were analyzed with ANOVA. Results were considered significant when p≤0.001. Pairwise comparisons among groups were adjusted using Bonferroni method to control for Type 1 error.

As shown in Table 1, compared to the Psychedelic Group, the Non-Drug Group was significantly older and more likely to be female, white, not Hispanic, college educated, married, and a resident of the United States, and had a higher household income.

The differences between the psychedelic and non-drug participants shown in Table 1 were generally true for each of the four psychedelic groups alone (Table 2, indicated by data in bold font). Table 2 also shows that, compared to the other psychedelic groups, the Ayahuasca Group was older at the time of the experience and survey, more likely to be female, college educated, married, not a U.S resident, and have a higher income. In contrast, the DMT Group was significantly younger at the time of the study than the other psychedelic groups. Open ended text responses from those in the DMT other than ayahuasca group indicated that this group was comprised primarily of those who smoked DMT; of the 606 DMT users, only 3 wrote comments suggesting an intranasal route of administration.

### Details of the encounter

As shown in Table 3, only about 20% of participants went into the experience with an intention of having an encounter experience. The Non-Drug Group was significantly more likely than the Psychedelic Group of being alone during the encounter (58% vs. 35%). All survey participants endorsed involvement of one or more senses during the encounter. For both groups, the primary senses engaged during the encounter were visual (48% vs. 75%), auditory (36% vs. 49%), bodily sensation/tactile (43% vs. 48%), and extrasensory (64% vs. 86%) for the Non-Drug and Psychedelic groups, respectively, with these differences being significant for all except tactile. Most participants (~65%), from both groups endorsed communication (i.e. an exchange of information with the entity). Participants from both groups endorsed similar rates of their having had an emotional response during the encounter (~90%), having ascertained a message, mission, or insight (~75%), or having acquired predictions about the future (~20%).

**Table 3:** Details of the encounter in the Non-Drug Group and combined Psychedelic Group^1^^,^^2^.

Questionnaire Item	Non-Drug Group (N = 809)	Psychedelic Group (N = 3476)
*Details of initiating the encounter (percentage endorsing the item)*		
Went into the experience with the intention of encountering that which was encountered	16%	22%
The encounter was initiated by that which was encountered (not by me)	55%	**45%**
Was alone (not with other people) at the time of the encounter	58%	**35%**
*Senses with which you interacted during the encounter (percentage endorsing the item)*		
Visual	48%	**75%**
Auditory (aural)	36%	**49%**
Bodily sensation/tactile (sense of touch)	43%	48%
Taste (gustatory)	3%	**10%**
Smell (olfactory)	6%	12%
Extrasensory	64%	**86%**
*Communication (percentage endorsing the item)*		
There was communication (1-way or 2-way exchange of information)	63%	67%
Communication was a 2-way exchange of information	22%	25%
Communication was a 1-way exchange of information (from it to you)	23%	25%
Communication was a 1-way exchange of information (from you to it)	4%	2%
Communication was visual (e.g. gestures)	15%	**25%**
Communication was verbal-auditory	26%	21%
Communication was somatic (e.g. touch/kinesthetic)	17%	14%
Communication was extrasensory-telepathic	45%	**60%**
*Immediate results of the encounter (percentage endorsing the item)*		
You had an emotional response during the encounter	91%	88%
That which was encountered had an emotional response during the encounter	23%	25%
You ascertained a message, task, mission, or insight from the encounter	78%	75%
You acquired predictions about the future	21%	24%

^1^ Within a row, bold font indicates significant difference from the Non-Drug Group.

^2^ Data are the percentage of the participants in the group that endorsed the items as positive. Statistical comparisons were adjusted for eight covariates (see Statistical section). Results were considered significant when p≤0.001. Estimated means and standard errors of the estimate are presented in Table A in S1 File.

As shown in Table 4, the pattern of differences between psychedelic and non-drug participants shown in Table 3 also occurred in each of the four psychedelic groups. Across the psychedelic groups, the Psilocybin and LSD groups did not significantly differ on any of these items. Likewise, the Ayahuasca and DMT groups differed on only 3 of the 21 items. The DMT Group tended to have the highest rates of endorsement among the drug groups and these differences were significantly higher than the Psilocybin and LSD groups on several sensory and communication items.

**Table 4:** Details of the encounter in the Non-Drug, psilocybin, LSD, ayahuasca, and DMT groups^1^^,^^2^.

Questionnaire Item	Non-Drug Group (N = 809)	Psilocybin Group (N = 1184)	LSD Group (N = 1251)	Ayahuasca Group (N = 435)	DMT Group (N = 606)
*Details of initiating the encounter (proportion endorsing the item)*					
Went into the experience with the intention of encountering that which was encountered	16%	20%^a^	18%^a^	**29%**^a,b^	**30%**^b^
The encounter was initiated by that which was encountered (not by me)	55%	**44%**^b^	**38%**^b^	55%^a^	56%^a^
Was alone (not with other people) at the time of the encounter	58%	**43%**^**a**^	**36%**^a^	**12%**^b^	**35%**^a^
*Senses with which you interacted during the encounter (percentage endorsing the item)*					
Visual	48%	**72%**^a^	**74%**^a^	**72%**^a,b^	**83%**^b^
Auditory (aural)	36%	45%^a^	49%^a,b^	**50%**^a,b^	**58%**^b^
Bodily sensation/tactile (sense of touch)	43%	46%^a^	50%^a^	47%^a^	48%^a^
Taste (gustatory)	3%	**8%**^a^	**11%**^a^	8%^a^	**11%**^a^
Smell (olfactory)	6%	10%^a^	13%^a^	11%^a^	13%^a^
Extrasensory	64%	**87%**^a^	**85%**^a^	**85%**^a^	**89%**^a^
*Communication (percentage endorsing the item)*					
There was communication (1-way or 2-way exchange of information)	63%	64%^a^	60%^a^	**80%**^b^	**80%**^b^
Communication was a 2-way exchange of information	22%	24%^a^	20%^a^	**40%**^b^	28%^a^
Communication was a 1-way exchange of information (from it to you)	23%	23%^a^	22%^a^	22%^a^	**38%**^b^
Communication was a 1-way exchange of information (from you to it)	4%	2%^a^	2%^a^	2%^a^	1%^a^
Communication was visual (e.g. gestures)	15%	23%^a,b^	21%^a^	**29%**^b,c^	**36%**^c^
Communication was verbal-auditory	26%	21%^a^	19%^a^	25%^a^	25%^a^
Communication was somatic (e.g. touch/kinesthetic)	17%	12%^a^	12%^a^	18%^a^	18%^a^
Communication was extrasensory-telepathic	45%	**57%**^a^	52%^a^	**71%**^b^	**75%**^b^
*Immediate results of the encounter (percentage endorsing the item)*					
You had an emotional response during the encounter	91%	88%^a^	89%^a^	86%^a^	89%^a^
That which was encountered had an emotional response during the encounter	23%	23%^a^	22%^a^	28%^a,b^	33%^b^
You ascertained a message, task, mission, or insight from the encounter	78%	74%^a^	73%^a^	82%^a^	74%^a^
You acquired predictions about the future	21%	24%^a^	25%^a^	27%^a^	21%^a^

^1^ Within a row, bold font indicates significant difference from the Non-Drug Group; for the drug groups, values not sharing a common letter are significantly different.

^2^ Data are the percentage of the participants in the group that endorsed the item as positive. Statistical comparisons were adjusted for eight covariates (see Statistical section). Pairwise comparisons were adjusted using Bonferroni method and results were considered significant when p≤0.001. Estimated means and standard errors of the estimate are presented in Table B in S1 File.

### Memory, realism, and mystical features of the encounter experience

As shown in Table 5, both groups provided high ratings of the vividness of their memories of the experience, with the Non-Drug Group having significantly higher ratings (92 vs. 76 of 100). Both groups provided relatively high similar ratings that the experience was more real than everyday normal reality. Table 5 also shows that factor and total scores on the Mystical Experience Questionnaire were uniformly high (≥0.70) in both groups, with the exception that the Transcendence of time and space factor was only intermediate in the Non-Drug Group. These scores were significantly higher in the Psychedelic Group than the Non-Drug Group, as was the percentage of participants in the group having a "complete" mystical experience (64% vs. 43%).

**Table 5:** Memory, realism, and mystical features of the encounter experience in the Non-Drug Group and combined Psychedelic Group^1^^,^^2^.

Questionnaire Item	Non-Drug Group (N = 809)	Psychedelic Group (N = 3476)
*Memory for encounter (ratings from 0 to 100)*		
Vividness of memories of the encounter	91.9 (14.2)	**76.2 (23.6)**
*Realism of the encounter (ratings from 0 to 100)*		
Superficial dream-like level of reality	25.0 (35.3)	**41.9 (37.7)**
Reality similar to everyday normal consciousness	54.5 (40.2)	**40.6 (36.0)**
More real than everyday normal consciousness	72.7 (36.3)	76.5 (32.2)
*Mystical Experience Questionnaire*: *Factor and total scores (proportion of maximum possible score)*		
Mystical factor	.73 (0.22)	**.81 (0.17)**
Positive mood factor	.78 (0.21)	**.80 (0.18)**
Transcendence of time and space factor	.54 (0.33)	**.73 (0.23)**
Ineffability factor	.77 (0.25)	**.85 (0.18)**
Total Score	.70 (0.21)	**.79 (0.15)**
*Mystical Experience Questionnaire*: *"Complete" mystical experience*		
Percentage of participants fulfilling criteria for complete experience	43%	**64%**

^1^ Within a row, bold font indicates significant difference from the Non-Drug Group.

^2^ For continuous measures, data are means and standard deviations. Dichotomous data for complete mystical experiences are percentage of participants in the group. Statistical comparisons for continuous and dichotomous data were adjusted for eight covariates (see Statistical section). Results were considered significant when p≤0.001. Estimated means and standard errors of the estimates are presented in Table C in S1 File.

Table 6 shows that the pattern of differences between psychedelic and non-drug participants shown in Table 5 occurred in each of the four psychedelic groups. The Psilocybin and LSD groups did not differ significantly on any of these measures. On measures of mystical experience, the DMT Group was significantly higher than the Psilocybin and LSD groups on Transcendence of time and space, Ineffability, Total score, and the percentage showing complete mystical experiences.

**Table 6:** Memory, realism, and mystical features of the encounter experience in the Non-Drug, psilocybin, LSD, ayahuasca, and DMT groups^1^^,^^2^.

Questionnaire Item	Non-Drug Group (N = 809)	Psilocybin Group (N = 1184)	LSD Group (N = 1251)	Ayahuasca Group (N = 435)	DMT Group (N = 606)
*Memory for the encounter (ratings from 0 to 100)*					
Vividness of memories of the encounter	91.9 (14.2)	**75.6 (23.3)**^a^	**76.6 (23.8)**^a^	**81.0 (21.2)**^a^	**72.9 (24.6)**^a^
*Realism of the encounter (ratings from 0 to 100)*					
Superficial dream-like level of reality	25.0 (35.3)	**42.7 (37.2)**^a^	**40.4 (37.3)**^a^	**37.1 (37.4)**^a^	**47.2 (38.8)**^a^
Reality similar to everyday normal consciousness	54.5 (40.2)	**42.1 (35.4)**^a^	**40.7 (35.9)**^a^	**42.6 (38.0)**^a^	**36.0 (35.7)**^a^
More real than everyday normal consciousness	72.7 (36.3)	74.7 (32.5)^a^	76.8 (32.5)^a^	79.4 (30.3)^a^	77.5 (32.2)^a^
*Mystical Experience Questionnaire*: *Factor and total scores (proportion of maximum possible score)*					
Mystical factor	.73 (0.22)	**.80 (0.17)**^a^	**.81 (0.18)**^a^	**.83 (0.16)**^a^	**.81 (0.17)**^a^
Positive mood factor	.78 (0.21)	.79 (0.18)^a^	.79 (0.19)^a^	.81 (0.17)^a^	.81 (0.18)^a^
Transcendence of time and space factor	.54 (0.33)	**.70 (0.24)**^a^	**.71 (0.24)**^a^	**.72 (0.22)**^a^	**.84 (0.19)**^b^
Ineffability factor	.77 (0.25)	**.84 (0.18)**^a^	**.84 (0.18)**^a^	**.84 (0.18)**^a,b^	**.88 (0.17)**^b^
Total Score	.70 (0.21)	**.78 (0.15)**^a^	**.79 (0.15)**^a^	**.80 (0.14)**^a,b^	**.82 (0.14)**^b^
*Mystical Experience Questionnaire*: *"Complete" mystical experience*					
Percentage of participants fulfilling criteria for complete experience	43%	**62%**^a^	**61%**^a^	**65%**^a,b^	**73%**^b^

^1^ Within a row, bold font indicates significant difference from the Non-Drug Group; for the drug groups, values not sharing a common letter are significantly different.

^2^ For continuous measures, data are means and standard deviations. Dichotomous data for complete mystical experiences are percentage of participants in the group. Statistical comparisons for continuous and dichotomous data were adjusted for eight covariates (see Statistical section). For both types of analyses, pairwise comparisons were adjusted using Bonferroni method and results were considered significant when p≤0.001. Estimated means and standard errors of the estimate are presented in Table D in S1 File.

### Interpretation of that which was encountered

Participants were asked to indicate which of four descriptors best described what was encountered. As shown in Table 7, the Non-Drug Group was significantly more likely than the Psychedelic Group to endorse encountering God (the God of your understanding) (41% vs. 18%) or an Emissary of God (18% vs. 9%). Conversely, endorsement of encountering Ultimate Reality was significantly more likely in the Psychedelic Group than the Non-Drug Group (55% vs. 26%, respectively). Rates of endorsement for Higher Power did not significantly differ between the Non-Drug Group and Psychedelic Group (15% vs 19%).

**Table 7:** Interpretation of that which was encountered in the Non-Drug Group and combined Psychedelic Group^1^^,^^2^^,^^3^.

Questionnaire Item	Non-Drug Group (N = 809)	Psychedelic Group (N = 3476)
*Best descriptor of that which was encountered (percentage endorsing the item)*		
God (the God of your understanding)	41%	**18%**
Ultimate Realty	26%	**55%**
Higher Power	15%	19%
An aspect or emissary of God (e.g. an angel)	18%	**9%**
*Attributes to that which was encountered (percentage endorsing the item)*^3^		
Benevolent (i.e. kind, compassionate, altruistic)	86%	**70%**
Intelligent	80%	78%
Sacred	81%	71%
Conscious (i.e. self-aware)	71%	68%
Eternal	70%	70%
All Knowing	66%	59%
Agency (e.g. could it affect outcomes, events, or material objects in this reality)	47%	**36%**
Petitionable (e.g. in response to prayer or petition, it might change events or circumstances)	32%	**18%**
Positively Judgmental (e.g. inclined toward strong approval or reward)	23%	29%
Negatively Judgmental (e.g. inclined toward strong disapproval or harsh punishment)	5%	6%
Malicious (i.e., unkind, cruel, vengeful)	1%	**9%**
*Additional interpretation of that which was encountered (percentage endorsing the item)*		
That which was encountered existed, as least in part, in some other dimension or reality	68%	69%
You were completely the same as that which was encountered	32%	**47%**
That which was encountered continued to exist after the encounter	74%	65%

^1^ Within a row, bold font indicates significant difference from the Non-Drug Group.

^2^ Data are the percentage of the participants in the group that endorsed the items as positive. Statistical comparisons were adjusted for eight covariates (see Statistical section). Results were considered significant when p≤0.001. Estimated means and standard errors of the estimate are presented in Table E in S1 File.

^3^ Response options for these questions were Yes, No, and I don’t know.

Interestingly, despite differences in the preferred descriptors of that which was encountered, there was a striking similarity in the relative percentages of each group that endorsed the 11 attributes of that which was encountered (Table 7). Furthermore, more than half of each group endorsed the attributes of benevolence, intelligence, sacredness, consciousness, being eternal, and being all-knowing. The Non-Drug Group was significantly more likely to endorse benevolence, agency, and being petitionable, and less likely to endorse being malicious than the Psychedelic Group. About 70% of both groups endorsed that that which was encountered existed, at least in part, in some other dimension or reality, and that which was encountered continued to exist after the encounter. Those participants who endorsed a given attribute as present then rated the degree to which that attribute applied on a 100-point scale (e.g. from "not at all" to "completely"). Mean ratings of the attributes of sacred, intelligent, benevolent, and conscious were ≥89 in both groups, with benevolent and sacred significantly higher in the Non-Drug Group. The only other attribute that was significantly different between groups was positively judgmental, with mean ratings of 87 and 77 in the Non-Drug and Psychedelic groups respectively.

Table 8 shows that the pattern of differences between Psychedelic and Non-Drug groups shown in Table 7 occurred in each of the four psychedelic groups. The Non-Drug Group endorsed having had an encounter with God (the God of your understanding) at a significantly higher rate than each of the four psychedelic groups alone and, conversely, endorsed having encountered Ultimate Reality at a significantly lower rate than the four psychedelic groups. With regard to the attributes of that which was encountered and the additional interpretation items, there were both similarities and differences among the drug groups (Table 8). The Psilocybin and LSD groups did not significantly differ on any of these items. Likewise, the Ayahuasca and DMT groups differed on only 2 of the 18 items. The Ayahuasca Group had significantly higher rates of endorsement than the Psilocybin and LSD groups of the positive attributes of that which was encountered of benevolence, intelligence, conscious, and being petitionable. For the 100-point ratings of the degree to which attributes applied, the only significant differences between the psychedelic groups was for the attribute of conscious, with DMT>LSD and Psilocybin, and Ayahuasca>LSD.

**Table 8:** Interpretation of that which was encountered in Non-Drug, psilocybin, LSD, ayahuasca, and DMT groups^1^^,^^2^^,^^3^.

Items	Non-Drug Group (N = 809)	Psilocybin Group (N = 1184)	LSD Group (N = 1251)	Ayahuasca Group (N = 435)	DMT Group (N = 606)
*Best descriptor of that which was encountered (percentage endorsing the item)*					
God (the God of your understanding)	41%	**16%**^a^	**19%**^a^	**21%**^a^	**16%**^a^
Ultimate Realty	26%	**57%**^a**,**b^	**59%**^b^	**46%**^a^	**48%**^a^
Higher Power	15%	19%^a,b^	16%^a^	21%^a,b^	25%^b^
An aspect or emissary of God (e.g. an angel)	18%	9%^a^	**6%**^a^	12%^a^	12%^a^
*Attributes to that which was encountered (percentage endorsing the item)*^3^					
Benevolent (i.e. kind, compassionate, altruistic)	86%	**66%**^b^	**66%**^b^	85%^a^	75%^a^
Intelligent	80%	73%^a^	73%^a^	**91%**^b^	**87%**^b^
Sacred	81%	71%^a,b^	68%^b^	80%^a^	69%^a,b^
Conscious (i.e. self-aware)	71%	62%^a^	64%^a^	**80%**^b^	77%^b^
Eternal	70%	70%^a^	72%^a^	75%^a^	63%^a^
All Knowing	66%	58%^a^	58%^a^	66%^a^	59%^a^
Agency (e.g. could it affect outcomes, events, or material objects in this reality)	47%	**34%**^a^	**38%**^a^	41%^a^	**31%**^a^
Petitionable (e.g. in response to prayer or petition, it might change events or circumstances)	32%	**17%**^b^	**17%**^b^	26%^a^	**16%**^b^
Positively Judgmental (e.g. inclined toward strong approval or reward)	23%	29%^a^	26%^a^	29%^a^	33%^a^
Negatively Judgmental (e.g. inclined toward strong disapproval or harsh punishment)	5%	8%^a^	8%^a^	6%^a^	10%^a^
Malicious (i.e., unkind, cruel, vengeful)	1%	**9%**^a^	**10%**^a^	**7%**^a^	**8%**^a^
*Additional interpretation of that which was encountered (percentage endorsing the item)*					
That which was encountered existed, as least in part, in some other dimension or reality	68%	65%^a^	66%^a,b^	**76%**^b,c^	**76%**^c^
You were completely the same as that which was encountered	32%	**46%**^a,b^	**52%**^b^	**44%**^a,b^	42%^a^
That which was encountered continued to exist after the encounter	74%	64%^a^	68%^a^	75%^a^	**53%**^b^

^1^ Within a row, bold font indicates significant difference from the Non-Drug Group; for the drug groups, values not sharing a common letter are significantly different.

^2^ Data are the percentage of the participants in the group that endorsed the items as positive. Statistical comparisons were adjusted for eight covariates (see Statistical section). Pairwise comparisons were adjusted using Bonferroni method and results were considered significant when p≤0.001. Estimated means and standard errors of the estimate are presented in Table F in S1 File.

^3^ Response options for these questions were Yes, No, and I don’t know.

### Comparison of encounter experience relative to other lifetime experiences

Participants were asked to rate several dimensions of their encounter experience relative to other experiences over their lifetimes. As shown in Table 9, ratings of personal meaning and spiritual significance were similar, with more than 74% of the Non-Drug and Psychedelic groups indicating the experience to be among the top 5 most meaningful and spiritually significant experiences of their lifetime, and 34% and 42%, respectively, indicating that the experience was the single most spiritually significant experience of their life. The percentage endorsement and relative ratings of psychological insight and psychological challenge were numerically lower than those for meaning and spiritual significance, with ratings but not percentage endorsement being significantly higher in the Psychedelic Group.

**Table 9:** Comparison of encounter experience relative to other lifetime experiences in the Non-Drug Group and combined Psychedelic Group^1^^,^^2^^,^^3^.

Questionnaire Item	Non-Drug Group (N = 809)	Psychedelic Group (N = 3476)
*Rating relative to other lifetime experiences (ratings from 1 to 8)*		
How personally meaningful was the encounter	6.85 (1.14)	6.91 (1.06)
How spiritually significant was the encounter	6.91 (1.30)	7.05 (1.31)
How personally psychologically insightful was the encounter	5.94 (2.19)	**6.53 (1.60)**
How psychologically challenging was the encounter	4.21 (2.79)	**5.27 (2.41)**
*Percentage rating the item as among the top 5 or single most of lifetime*		
How personally meaningful was the encounter	74%	78%
How spiritually significant was the encounter	78%	83%
How personally psychologically insight was the encounter	58%	67%
How psychologically challenging was the encounter	32%	44%
*Percentage rating the item as the single most of lifetime*		
How personally meaningful was the encounter	28%	27%
How spiritually significant was the encounter	34%	42%
How personally psychologically insight was the encounter	22%	27%
How psychologically challenging was the encounter	12%	17%

^1^ Within a row, bold font indicates significant difference from the Non-Drug Group.

^2^ For continuous measures, data are means and standard deviations. Dichotomous data are the percentage of the participants in the group that endorsed the item as positive. Statistical comparisons for continuous and dichotomous data were adjusted for eight covariates (see Statistical section). Results were considered significant when p≤0.001. Estimated means and standard errors of the estimates are presented in Table G in S1 File.

^3^ Rating options ranged from 1 = no more than routine, everyday experience; 5 = similar to experiences that occur on average once every 5 years; 6 = among the 10 most in my life; 7 = among the 5 most of my life; 8 = the single most of my life.

Table 10 shows that the pattern of similarities and differences between Psychedelic and Non-Drug groups shown in Table 9 occurred in each of the four psychedelic groups. The Psilocybin, LSD, and DMT groups did not significantly differ on any of these measures. The Ayahuasca Group was usually numerically higher than the other groups and sometimes significantly higher than the Psilocybin and LSD groups.

**Table 10:** Comparison of encounter experience relative to other lifetime experiences in the Non-Drug, psilocybin, LSD, ayahuasca, and DMT groups^1^^,^^2^^,^^3^.

Questionnaire Item	Non-Drug Group (N = 809)	Psilocybin Group (N = 1184)	LSD Group (N = 1251)	Ayahuasca Group (N = 435)	DMT Group (N = 606)
*Rating relative to other lifetime experiences (ratings from 1 to 8)*					
How personally meaningful was the encounter	6.85 (1.14)	6.83 (1.05)^a^	6.87 (1.10)^a,b^	7.13 (0.88)^b^	6.97 (1.07)^a,b^
How spiritually significant was the encounter	6.91 (1.30)	6.99 (1.43)^a^	6.97 (1.41)^a^	**7.30 (0.88)**^b^	7.14 (1.26)^a,b^
How personally psychologically insightful was the encounter	5.94 (2.19)	**6.49 (1.52)**^a^	**6.48 (1.72)**^a^	**6.77 (1.43)**^a^	**6.54 (1.62)**^a^
How psychologically challenging was the encounter	4.21 (2.79)	5.13 (2.41)^a^	**5.26 (2.46)**^a,b^	**5.61 (2.25)**^b^	**5.33 (2.42)**^a,b^
*Proportion rating the item as among the top 5 or single most of lifetime*					
How personally meaningful was the encounter	74%	75%^a^	77%^a,b^	83%^b^	81%^a,b^
How spiritually significant was the encounter	78%	81%^a^	81%^a^	**89%**^b^	85%^a,b^
How personally psychologically insight was the encounter	58%	65%^a^	68%^a^	71%^a^	67%^a^
How psychologically challenging was the encounter	32%	42%^a^	44%^a^	46%^a^	47%^a^
*Proportion rating the item as the single most of lifetime*					
How personally meaningful was the encounter	28%	**23%**^a^	26%^a^	36%^b^	30%^a,b^
How spiritually significant was the encounter	34%	41%^a^	40%^a^	47%^a^	47%^a^
How personally psychologically insight was the encounter	22%	24%^a^	28%^a,b^	34%^b^	29%^a,b^
How psychologically challenging was the encounter	12%	14%^a^	18%^a,b^	21%^b^	18%^a,b^

^1^ Within a row, bold font indicates significant difference from the Non-Drug Group; for the drug groups, values not sharing a common letter are significantly different.

^2^ For continuous measures, data are means and standard deviations. Dichotomous data are percentage of participants in the group that endorsed the item as positive. Statistical comparisons for continuous and dichotomous data were adjusted for eight covariates (see Statistical section). For both types of analyses, pairwise comparisons were adjusted using Bonferroni method. Results were considered significant when p≤0.001. Estimated means and standard errors of the estimate are presented in Table H in S1 File.

^3^ Rating options ranged from 1 = no more than routine, everyday experience; 5 = similar to experiences that occur on average once every 5 years; 6 = among the 10 most in my life; 7 = among the 5 most of my life; 8 = the single most of my life.

### Persisting changes attributed to the encounter

The Non-Drug and Psychedelic groups had largely similar responses to a series of questions probing persisting changes that they attributed to the encounter experience. As shown in Table 11, both groups rated positive, desirable changes, generally of moderate strength (mean = 2.0, see table footnote) across nine persisting effect items. The only significant difference was that rating of positive changes in spiritual awareness in everyday life was greater in the Non-Drug Group. Furthermore, the majority of both groups endorsed a desirable change in contemplative, prayer, or meditation practice, a desirable change in understanding religious traditions of others, and decreased fear of death. The Psychedelic Group was significantly more likely to endorse a decreased fear of death than the Non-Drug Group (70% vs. 57%).

**Table 11:** Persisting changes attributed to the encounter in the Non-Drug Group and combined Psychedelic Group^1^^,^^2^.

Questionnaire Item	Non-Drug Group (N = 809)	Psychedelic Group (N = 3476)
*Persisting changes attributed to the encounter experience (ratings from -3 to +3*)^3^		
Personal sense of well-being or life satisfaction	2.38 (0.99)	2.21 (1.03)
Your life’s purpose	2.16 (1.08)	1.97 (1.14)
Your life’s meaning	2.21 (1.07)	1.99 (1.15)
Your social relationships (e.g. family, friends, neighbors, strangers etc.)	1.76 (1.31)	1.67 (1.25)
Your spiritual awareness in everyday life	2.44 (0.86)	**2.16 (0.99)**
Your attitudes about life	2.27 (1.00)	2.18 (1.00)
Your attitudes about self	2.16 (1.07)	2.06 (1.06)
Your mood	1.54 (1.21)	1.53 (1.19)
Your behavior	1.77 (1.15)	1.58 (1.14)
*Persisting changes attributed to encounter experience (percentage endorsing the item*)		
Desirable change in contemplative, prayer, or meditation practice	89%	85%
Undesirable change in contemplative, prayer, or meditation practice	1%	1%
Desirable change in understanding religious or spiritual traditions other than your own	79%	86%
Undesirable change in understanding religious or spiritual traditions other than your own	1%	2%
Decreased fear of death	57%	**70%**
Increased fear of death	1%	3%

^1^ Within a row, bold font indicates significant difference from the Non-Drug Group.

^2^ For continuous measures, data are means and standard deviations. Dichotomous data are the percentage of the participants in the group that endorsed the item as positive. Statistical comparisons for continuous and dichotomous data were adjusted for eight covariates (see Statistical section). Results were considered significant when p≤0.001. Estimated means and standard errors of the estimates are presented in Table I in S1 File.

^3^ Rating options ranged from -3 = Strong negative change that I consider undesirable to +2 Moderate positive change that I consider desirable and +3 = Strong positive change that I consider desirable.

Table 12 shows that the pattern of similarities and differences between Non-Drug and Psychedelic groups shown in Table 11 occurred in each of the four psychedelic groups. The Psilocybin, LSD, and DMT groups did not significantly differ on any of these measures, with the exception that a larger proportion of the DMT Group endorsed a decreased fear of death. The Ayahuasca Group had significantly higher ratings than the Psilocybin and LSD groups on positive changes in life satisfaction, social relationships, spiritual awareness in everyday life, attitudes about life, attitudes about self, mood, and behavior.

**Table 12:** Persisting changes attributed to the encounter in the Non-Drug, psilocybin, LSD, ayahuasca, and DMT groups^1^^,^^2^.

Items	Non-Drug Group (N = 809)	Psilocybin Group (N = 1184)	LSD Group (N = 1251)	Ayahuasca Group (N = 435)	DMT Group (N = 606)
*Persisting changes attributed to the encounter experience (ratings from -3 to +3)*^3^					
Personal sense of well-being or life satisfaction	2.38 (0.99)	2.17 (1.03)^a^	2.14 (1.10)^a^	2.46 (0.84)^b^	2.26 (0.97)^a,b^
Your life’s purpose	2.16 (1.08)	1.94 (1.13)^a^	1.92 (1.20)^a^	2.16 (0.98)^a^	1.99 (1.14)^a^
Your life’s meaning	2.21 (1.07)	1.99 (1.15)^a,b^	**1.90 (1.21)**^b^	2.20 (1.01)^a^	2.02 (1.12)^a,b^
Your social relationships (e.g. family, friends, neighbors, strangers etc.)	1.76 (1.31)	1.68 (1.21)^a^	1.54 (1.31)^**a**^	2.03 (1.09)^b^	1.69 (1.25)^a^
Your spiritual awareness in everyday life	2.44 (0.86)	**2.15 (0.96)**^b^	**2.08 (1.07)**^b^	2.36 (0.85)^a^	2.21 (0.95)^a,b^
Your attitudes about life	2.27 (1.00)	2.14 (1.01)^a^	2.14 (1.04)^a^	2.36 (0.84)^b^	2.24 (0.98)^a,b^
Your attitudes about self	2.16 (1.07)	2.03 (1.06)^a^	2.00 (1.12)^a^	2.30 (0.84)^b^	2.10 (1.03)^a,b^
Your mood	1.54 (1.21)	1.52 (1.19)^a^	1.39 (1.24)^a^	**1.82 (1.04)**^b^	1.63 (1.16)^a,b^
Your behavior	1.77 (1.15)	1.56 (1.15)^b^	**1.47 (1.18)**^b^	1.90 (0.96)^a^	1.64 (1.13)^a,b^
*Persisting changes attributed to the encounter experience (proportion endorsing the item)*					
Desirable change in contemplative, prayer, or meditation practice	89%	86%^a^	83%^a^	88%^a^	85%^a^
Undesirable change in contemplative, prayer, or meditation practice	1%	1%^a^	1%^a^	1%^a^	1%^a^
Desirable change in understanding religious or spiritual traditions other than your own	79%	85%^a^	86%^a^	87%^a^	86%^a^
Undesirable change in understanding religious or spiritual traditions other than your own	1%	3%^a^	1%^a^	1%^a^	2%^a^
Decreased fear of death	57%	70%^a,b^	67%^a^	**73%**^a,b^	**77%**^b^
Increased fear of death	1%	3%^a^	4%^a^	3%^a^	3%^a^

^1^ Within a row, bold font indicates significant difference from the Non-Drug Group; for the drug groups, values not sharing a common letter are significantly different.

^2^ For continuous measures, data are means and standard deviations. Dichotomous data are percentage of participants in the group that endorsed the items as positive. Statistical comparisons for continuous and dichotomous data were adjusted for eight covariates (see Statistical section). For both types of analyses, pairwise comparisons were adjusted using Bonferroni method. Results were considered significant when p≤0.001. Estimated means and standard errors of the estimate are presented in Table J in S1 File.

^3^ Rating options ranged from -3 = Strong negative change that I consider undesirable to +2 Moderate positive change that I consider desirable and +3 = Strong positive change that I consider desirable.

### Changes in identification as atheist and monotheist

As rated retrospectively, before the encounter experience, the Non-Drug Group, compared to the Psychedelic Group, was less likely to identify their religious orientation as atheist (3% vs. 21%) or other (50% vs. 67%), but more likely to identify as a monotheist (47% vs. 12%) (Table 13). In both groups, identification as atheist decreased significantly from before to after the experience (3% to 1% and 21% to 8%, respectively) (z-test of proportions, p≤0.05 for both groups). The proportion of participants in each group that identified as atheist before the encounter but no longer identified as atheist after the encounter (74% and 67%, respectively) was not significantly different. In the Psychedelic Group, identification as monotheist significantly decreased and identification as Other significantly increased from before to after the experience (p≤0.05). The proportion of the Non-Drug Group identifying as monotheist or Other did not differ significantly from before to after the experience.

**Table 13:** Religious orientation before and after the encounter experience for Non-Drug Group and the combined Psychedelic Group^1^^,^^2^^,^^3^.

Measure	Non-Drug Group (N = 809)	Psychedelic Group (N = 3476)
Identification as atheist (percentage of group)		
Before the experience	3%	**21%**
After the experience	1%	**8%**
Identification with major monotheistic tradition		
Before the experience	47%	**12%**
After the experience	41%	**7%**
Identification as Other (not atheist or major monotheistic tradition)		
Before the experience	50%	**67%**
After the experience	59%	**85%**

^1^ Within a row, bold font indicates significant difference from the Non-Drug Group.

^2^ Data are the percentage of the participants in the group that endorsed identification with the religious orientation. Statistical comparisons between groups were conducted with Chi Square tests. Pairwise comparisons between groups for each of the religious affiliation categories were conducted with z tests for independent proportions with Bonferroni adjustment (p≤0.05). In both groups, identification as atheist decreased significantly from before to after the experience (p≤0.05, z-tests for dependent proportions with Bonferroni adjustment).

^3^ Participants were asked to select the best descriptor from among 24 descriptors provided to designate their religious orientation immediately before the encounter experience and again after the experience. For analysis, data are expressed in three categories: atheist (those choosing the atheist descriptor); monotheist (those choosing Christian, Jewish or Islam descriptors), or other.

Table 14 shows that the pattern of differences between Non-Drug and Psychedelic groups and between before vs. after the experience shown in Table 13 occurred in each of the four psychedelic groups. As with the Non-Drug Group, identification as atheist decreased significantly from before to after the experience in each of the four psychedelic groups (z-tests of proportions, p≤0.05). The proportion identifying as monotheist decreased significantly in the Psilocybin and LSD groups, and the proportion identifying as other increased significantly in the Psilocybin, LSD, and DMT groups (p≤0.05).

**Table 14:** Religious orientation before and after the encounter experience for Non-Drug, psilocybin, LSD, ayahuasca, and DMT groups^1^^,^^2^^,^^3^.

Measure	Non-Drug Group (N = 809)	Psilocybin Group (N = 1184)	LSD Group (N = 1251)	Ayahuasca Group (N = 435)	DMT Group (N = 606)
Identification as atheist (percentage of group)					
Before the experience	3%	**21%**^b^	**22%**^b^	**12%**^a^	**25%**^b^
After the experience	1%	**9%**^b^	**9%**^b^	3%^a^	**7%**^a,b^
Identification with major monotheistic tradition					
Before the experience	47%	**12%**^**a,b**^	**15%**^**a**^	**9%**^**b,c**^	**7%**^**c**^
After the experience	41%	**8%**^**a,b**^	**9%**^**a**^	**6%**^**a,b**^	**5%**^**b**^
Identification as Other (not atheist or major monotheistic tradition)					
Before the experience	50%	**68%**^a^	**62%**^a^	**80%**^b^	**68%**^a^
After the experience	59%	**84%**^a,b^	**82%**^a^	**91%**^c^	**88%**^b,c^

^1^ Within a row, bold font indicates significant difference from the Non-Drug Group; for the drug groups, values not sharing a common letter are significantly different.

^2^ Data are the percentage of the participants in the group that endorsed identification with the religious orientation. Statistical comparisons between groups were conducted with Chi Square tests. Pairwise comparisons between groups for each of the religious affiliation categories were conducted with z tests for independent proportions with Bonferroni adjustment (p≤0.05). In all five groups, identification as atheist decreased significantly from before to after the experience (p≤0.05, z-tests for dependent proportions with Bonferroni adjustment).

^3^ See Table 13 for explanation of religious orientation categories.

## Discussion

This cross-sectional internet survey study with 4,285 participants is the first study to provide a direct and detailed comparison of naturally occurring (non-drug) and psychedelic-occasioned experiences that participants interpreted as an encounter with God (using any of four descriptors of such experiences). The study also provides new information about the characteristics and consequences of such experiences and permits comparison of experiences among those who consumed psilocybin, LSD, ayahuasca, or DMT.

Because of the large number of outcome measures and complexity the results, this Discussion section will first summarize the most salient similarities and differences between the non-drug and the psychedelic-occasioned experiences followed by a summary of comparisons among the four psychedelic groups.

### Similarities and differences between Non-Drug and psychedelic-occasioned experiences

Despite a few demographic differences (e.g. age, sex, country of residence), there were striking similarities in the details and consequences of the encounter experiences between the Non-Drug and Psychedelic groups, many of which are consistent with numerous historical descriptions of naturally occurring God encounter and mystical experiences [1,3]. In both groups, the encounter experiences were largely unbidden, with only about one in five participants indicating they had an intention for such an experience. All participants reported one or more senses being involved, with extrasensory, visual, auditory, and tactile senses being the most frequently endorsed. The majority as well as similar proportions of both groups reported communication (i.e. an exchange of information with that which was encountered), having a personal emotional response during the encounter, and having ascertained a message, mission, or insight, while only about one in five reported having acquired predictions about the future or that which was encountered having an emotional response during the encounter. Both groups provided moderately high ratings on the vividness of their memories of the encounter, that the experience seemed more real than everyday consciousness, and on the total score and most subscales of the Mystical Experience Questionnaire. Likewise, similar high proportions of the two groups endorsed a range of qualities attributed to that which was encountered, with the majority endorsing benevolent, intelligent, sacred, conscious, eternal and all knowing, but fewer than one in ten endorsing negatively judgmental or malicious. The majority of both groups endorsed that that which was encountered existed, at least in part, in some other reality and that it continued to exist after the encounter. About three-quarters or more of both groups indicated that the encounter was among the 5 most personally meaningful and spiritually significant experiences of their lifetimes, with about one in three indicating that it was the single-most such experience. With regard to persisting changes attributed to the experience, most participants in both groups endorsed desirable change in contemplative, prayer, or meditation practice and in understanding religious or spiritual traditions other than their own, and both groups had moderate to strong mean ratings of desirable changes in life satisfaction, purpose, meaning, spiritual awareness in everyday life, attitudes about life and self.

Despite these many similarities, there were some notable differences in details and consequences of the encounter experiences between the Non-Drug and Psychedelic groups. To emphasize the most robust differences between groups, this discussion will focus on significant differences (p≤0.001) in proportions of the two groups with the additional requirement that the difference was >10%. Compared to the Psychedelic Group, the Non-Drug Group was more likely to be alone at the time of the experience (58% vs. 35%) and less likely to endorse visual, auditory, or extrasensory senses being involved. Interestingly, the Non-Drug Group was more than twice as likely to endorse God (the God of your understanding) as the best descriptor of that which was encountered (41% vs. 18%), but less than half as likely to endorse the descriptor Ultimate Reality (26% vs. 55%). Consistent with the most common attributes of "God" in monotheistic traditions, the Non-Drug Group was significantly more likely to endorse that which was encountered had agency (could affect events in this reality) and was petitionable (responsive to prayer or petition), and less likely to endorse that the participant was the same as that which was encountered. The Psychedelic Group was more likely to endorse decreased fear of death.

Both groups showed moderately high scores on the Mystical Experience Questionnaire (MEQ-30). The Psychedelic Group, however, was significantly higher than the Non-Drug Group in total scores, each of the four factor scores, and proportion of the group fulfilling *a priori* criteria for having had a "complete" mystical experience (43% vs. 64%). It seems likely that the higher MEQ-30 scores in the Psychedelic Group may be due in part to the fact that the MEQ-30 was developed and validated to assess such experiences occasioned by psilocybin [29,30], and therefore may have more sensitivity to psychedelic experiences. These findings indicate that theistically interpreted, naturally occurring God encounter experiences may fulfill Stace’s [4] criteria for mystical experience that make no reference to God. The findings also suggest that the MEQ-30 may be useful for assessing naturally occurring spiritual and God encounter experiences.

**Fig 1:**
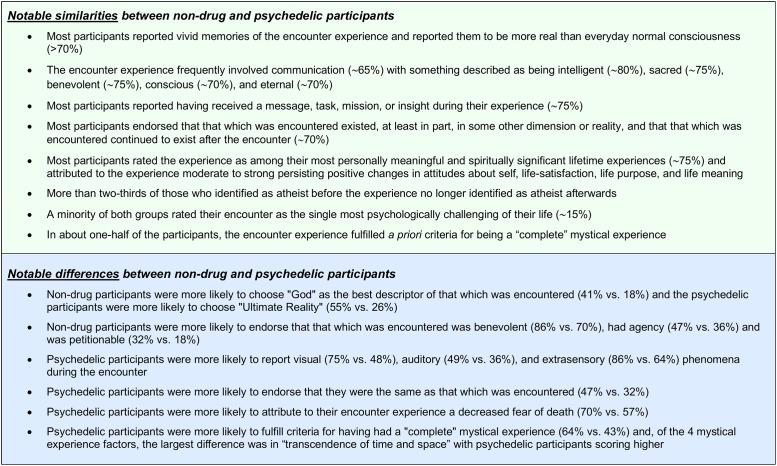
Similarities and differences in God encounter experiences between Non-Drug and psychedelic participants. Summary of notable similarities and differences in details, features, interpretation, and persisting changes of God encounter experiences between the Non-Drug Group (naturally occurring experiences) and the combined Psychedelic Group (psychedelic-occasioned experiences). Approximate percentages of the participants in the groups that endorsed the item are presented for some items; actual percentages are presented in Tables 3–11 and Results section.

Fig 1 presents a summary of the most notable similarities and differences between the Non-Drug Group and the Psychedelic Group.

A recent cross-sectional internet survey study by Yaden and colleagues [37] examined religious, spiritual, and mystical experiences (RSMEs) and psychedelic use. In their study, a group of participants reporting that they had used one or more psychedelic substance that had influenced their lifetime RSMEs were compared to a group reporting they had not used a psychedelic that influenced their RSMEs. The study showed that the psychedelic group attributed to their lifetime RSMEs a greater sense of purpose and spirituality and a reduced fear of death. Consistent with Yaden et al., psychedelic users in the present study were more likely to endorse decreased fear of death. In contrast to Yaden et al., in the present study, the great majority of items assessing persisting changes attributed to the encounter experience were not different between the psychedelic and nonpsychedelic users (Table 11) and psychedelic users rated their persisting spiritual awareness in everyday life significantly *lower* than nonpsychedelic users. Although the Yaden et al. study and the present study both focus on the effects of psychedelic substances on spiritual experiences, there are important differences in methods that could partially explain these inconsistencies. Notably, the focus of the Yaden et al. study was on broadly described lifetime religious, spiritual, and mystical experiences in contrast to the present study which focused much more narrowly on a single experience of an encounter with something that might be called God, Higher Power, Ultimate Reality, or an Aspect or Emissary of God. In further contrast to the present study, Yaden and colleagues did not assess whether the RSMEs occurred on the same occasions that the psychedelic substances were taken, did not exclude the possible use of non-psychedelic drugs during the time of the RSMEs, and had much smaller sample sizes (≈330 and ≈330 vs. 809 and 3476).

### Similarities and differences among different psychedelics

#### Psilocybin and LSD groups were very similar

Except for some small but significant differences in age and years since the experience, the Psilocybin and LSD groups were not significantly different on any of the 76 items assessing the details and consequences of the encounter experience. This finding is interesting because, although psilocybin and LSD are both classic psychedelics whose primary effects are mediated at the 5HT_2A_ receptor, they have different molecular structures, profiles of receptor activity, durations of action, with likely differences in functional potency and selectivity (e.g. [38]).

#### Ayahuasca Group compared to the other psychedelic groups

Demographically, the Ayahuasca Group was the most unique of the psychedelic groups, being more likely to be older, female, college educated, married, and not a U.S. resident, and less likely to be atheist. These differences and the finding that the ayahuasca users were significantly less likely to have been alone at the time of the experience are consistent with ayahuasca being used in structured group settings for religious or spiritual purposes throughout the world [23]. The Ayahuasca Group was more likely to endorse having had communication with that which was encountered than did the Psilocybin and LSD groups. With regard to attributes of that which was encountered, the Ayahuasca Group tended to have the highest rates of endorsement of positive attributes of that which was encountered, with these being significantly higher than psilocybin and LSD for benevolence, intelligence, conscious, and being petitionable. Likewise, with regard to comparisons to other lifetime experiences and persisting changes, the Ayahuasca Group generally had the numerically highest ratings or highest rates of endorsement on questions indicating positive outcomes, with these being significantly higher than psilocybin and LSD for being spiritually significant and for increasing life satisfaction, social relationships, spiritual awareness in everyday life, attitudes about life and self, mood, and behavior. Although demographic differences between groups were adjusted statistically, detailed information about context of use was not obtained and, thus, it is not possible to determine the extent to which the differences in positive attributes to that encountered and to positive attributions to the experience and its consequences were due to the common use of ayahuasca in a structured religious/spiritual group context [23,39]. However, this is an important consideration because the potent influence of both psychological set and physical setting on the effects of classic psychedelics is well-known to researchers and practitioners who work with these compounds [40–42].

#### DMT Group compared to the Ayahuasca Group

As described above, the demographics of the DMT Group differed from the Ayahuasca Group and the ayahuasca users were less likely to be alone at the time of the experience. However, across the other 76 items assessing the details and consequences of the encounter experience, there were only a few differences. Notably, and consistent with the structured group religious use of ayahuasca [23,39], the DMT Group had significantly lower positive changes in their social relationships and were less likely to endorse that that which was encountered was petitionable or continued to exist after the encounter. DMT users also were more likely to endorse that communication was 1-way (from it to you) and less likely to endorse that communication was 2-way.

#### DMT Group compared to the psilocybin and LSD groups

Demographically, the DMT Group was similar to the Psilocybin and LSD groups except for being younger and having had the experience more recently. Despite the demographic similarities, the DMT Group differed significantly from the Psilocybin and LSD groups on 16 of 76 items assessing details and consequences of the experience. The DMT Group was more likely than the Psilocybin and LSD groups to have gone into the experience with the intention of an encounter, the encounter was more likely to have been initiated by the other, 1-way or 2-way communication was more likely to have occurred, the communication was more likely to be visual or extrasensory, and that which was encountered was more likely to be benevolent, intelligent, conscious, and to have existed in some other dimension but was less likely to continue to exist after the encounter. Compared to the Psilocybin and LSD groups, the DMT Group had significantly higher total scores on the Mystical Experience Questionnaire, with higher scores on ineffability and transcendence of time and space factors, and with a greater proportion of the group fulfilling criteria for a complete mystical experience. This survey cannot distinguish whether these differences in DMT experience from Psilocybin and LSD reflect true pharmacological differences versus differences in expectancy and context. It is plausible that popular beliefs about DMT effects, with special interest in DMT-occasioned entity encounter experiences, may have biased DMT users toward having such experiences [5, 43–45] (www.dmt-nexus.me).

Several of the findings described above are consistent with the conclusion that *N*,*N*-dimethyltryptamine accounts both for similarities between the DMT and Ayahuasca groups as well as the differences of each of these groups from the psilocybin and LSD groups. Although ayahuasca is an admixture of plants, *N*,*N*-dimethyltryptamine is considered to be the principal psychedelic component [46], as it is for those who use DMT alone. The overall profile of effects with the DMT Group was most similar to the Ayahuasca Group despite differences in demographics, popular beliefs about expected effects, and contexts of administration. Furthermore, DMT (other than in ayahuasca) is most commonly smoked, thus having a very rapid onset and short duration of effects [47], in contrast to ayahuasca which is ingested orally with a slower onset and longer duration of action [46]. Taken together, these results suggest that *N*,*N*-dimethyltryptamine produces robust effects across a wide range of conditions. Furthermore, the observation that significant differences or direction of differences from the Psilocybin and LSD groups were generally similar in the DMT and Ayahuasca groups suggests that *N*,*N*-dimethyltryptamine produces a unique profile of effects that is phenomenologically distinct from two widely used classic psychedelics (psilocybin and LSD), which were indistinguishable on all measures assessed in this survey.

### Changes in identification as atheist from before to after the experience

An interesting finding of the present study was that, in the Non-Drug Group and each of the psychedelic groups, most of those who identified their religious affiliation as atheist before the experience no longer identified as atheist after the encounter, with this difference being significant in all groups. This outcome is consistent with sudden religious conversion experiences that are well-described in the psychology of religion literature [1,6 (chapter 8)], with Paul’s experience of encountering Jesus on the road to Damascus as the prototype. An important future direction of research with psychedelic drugs will be to extend prospective research on psychedelic drug-occasioned experiences [26–28] to assess possible changes in religious orientation or affiliation including identification as atheist.

### Encounter experiences are not infrequently psychologically challenging

Although most participants rated the encounter as among the most personally meaningful and spiritually significant experiences of their lives, about one-third rated the experience as among the 5 most psychologically challenging experiences of their lives, with about 15% indicating that it was the single most psychologically challenging experience of their lifetime. That such experiences may be both attractive and extremely difficult is consistent with the classic description of the dual nature of encounters with the "Holy" both as "mysterium tremendum" (referring to its awfulness and absolute overpoweringness) and "mysterium fascinans" (referring to its fascinating and attractive nature) by the theologian Rudolf Otto [48]. Likewise, that psychedelic experiences can involve both positive emotion including transcendence as well as highly distressing feelings such as fear and insanity have been well-documented [29,49,50].

### Can psychedelic drugs occasion genuine God encounter experiences?

Although some scholars of religion have argued on conceptual grounds that drug-occasioned experiences are not genuine religious experiences [32–34], Stace [4] and Smith [35,51] counter with the Principal of Causal Indifference, which asserts that if two experiences are phenomenologically indistinguishable, it cannot be concluded that one is genuine but the other is not. Although there are both similarities and differences in the God encounter experiences described by the Non-Drug and Psychedelic groups, the most robust generality across a wide range of questions is that the descriptive details, interpretation, and consequences of these experiences are markedly similar. The findings that the preferred descriptor of that which was encountered was "God" in the Non-Drug Group, but "Ultimate Reality" in the Psychedelic Group suggest that such labels may reflect differences in semantics and conceptual interpretation rather than phenomenological or functional differences in the experience.

It should be noted that neither descriptive studies of such experiences, no matter how detailed, nor the emerging science of neurotheology, no matter how strong the associations demonstrated between brain processes and religious experience, can definitively address ontological claims about the existence of God [5,52,53, 54]. We acknowledge that contentious issues arise from attempting to draw ontological conclusions about participants’ phenomenological experiences of "God" or "Ultimate Reality," which some believe to be beyond ordinary material reality/consciousness [55–56]. Such conceptual issues have been discussed at length by scholars of the psychology of religion who routinely use empirical methods in the study of religious, spiritual, and mystical experiences [6].

### Study strengths and limitations

The methodological strengths of this study include the detailed information assessed about a single experience in a large sample, exclusion from the Non-Drug Group of anyone who reported ever in their lifetime having had a God encounter experience after taking any psychoactive drug, exclusion from the Psychedelic Group of those whose experience occurred after taking multiple substances, and statistical adjustment for demographic differences between groups. However, there are a number of limitations of this study. One limitation is that the data are based entirely on self-reports collected retrospectively, often years after the experience occurred. Self-report is limited by social desirability or other implicit biases. For example, participants may have been more willing to provide affirmative responses to our survey questions because of their belief, whether accurate or not, that we may have wanted such responses. Although the majority of participants indicated that they had vivid memories, the very long delay between the experience and completing the questionnaire (on average over a decade) raises further concerns about whether these memories may have changed over time. Further study limitations include that the survey was time-consuming (averaging 50 minutes), uncompensated, and anonymous, which could have contributed to sample selection bias. On the other hand, these features also suggest that participants were highly motivated to provide detailed information about these experiences which they considered to be among the most meaningful of their lives. A related study limitation is that we do not know how representative the study samples are of the larger populations of individuals who may have had such experiences. Although the demographic characteristics of the Psychedelic Group were quite similar to those of past internet surveys of mystical-type and adverse experiences after psilocybin use [29,49], it is notable that only 1% of both the Non-Drug and Psychedelic Groups were Black/African-American, which would appear to significantly underrepresent this racial group. Future research should address this limitation by specifically recruiting individuals from a variety of diverse backgrounds to better understand these phenomena among non-White participants.

## Conclusions

This is the first study to provide a detailed comparison of naturally occurring (non-drug) and psychedelic-occasioned experiences that participants frequently interpreted as an encounter with God or Ultimate Reality. Although there are interesting differences between non-drug and psychedelic experiences, as well as between experiences associated with four different psychedelic drugs (psilocybin, LSD, ayahuasca, and DMT), the similarities among these groups are striking. Participants reported vivid memories of these encounter experiences which frequently involved communication with something most often described as God or Ultimate Reality and having the attributes of being conscious, benevolent, intelligent, sacred, eternal, and all-knowing. The encounter experience fulfilled *a priori* criteria for being a complete mystical experience in about half of the participants. Similar to mystical-type experiences, which are often defined without reference encountering a sentient other, these experiences were rated as among the most personally meaningful and spiritually significant lifetime experiences, with persisting moderate to strong positive changes in attitudes about self, life satisfaction, life purpose, and life meaning that participants attributed to these experiences. Future exploration of biological and psychological predisposing factors and the phenomenological and neural correlates of both the acute and persisting effects of such experiences may provide a deeper understanding of religious and spiritual beliefs that have been integral to shaping human cultures since time immemorial.

## Supporting information

S1 File Supporting information tables A, B, C, D, E, F, G, H, I, and J provide estimated means and standard errors of the estimate for data presented in the published manuscript in Tables 3, 4, 5, 6, 7, 8, 9, 10, 11 and 12, respectively. (PDF)

S2 File Questionnaire of naturally occurring (i.e. non-drug) God encounter experiences. (PDF)

S3 File Questionnaire of God encounter experiences occasioned by classic psychedelics. (PDF)
