# Supplementary material for: Survey of subjective "God encounter experiences": Comparisons among naturally occurring experiences and those occasioned by the classic psychedelics psilocybin, LSD, ayahuasca, or DMT
Source: PLoS One. 2019 Apr 23;14(4):e0214377. doi: 10.1371/journal.pone.0214377 (PMC6478303; doi:10.1371/journal.pone.0214377)
Supplement: S1 File — (PDF) [file pone.0214377.s001.pdf]

Supporting Information

S1 File. Supporting Information Tables A, B, C, D, E, F, G, H, I, and J provide estimated means and standard errors of the estimate for data presented in the published manuscript in Tables 3, 4, 5, 6, 7, 8, 9, 10, 11, and 12, respectively.

From: Griffiths, R.R., Hurwitz, E.S., Davis, A.K., Johnson, M.W., & Jesse, R. Survey of subjective "God encounter experiences": Comparisons among naturally occurring experiences and those occasioned by the classic psychedelics psilocybin, LSD, ayahuasca, or DMT. PLOS One

Supporting Information Table A: Details of the encounter in the Non-Drug group and combined Psychedelic Group. Data are estimated means and standard errors of the estimate for data from manuscript Table 3[1,2]

Questionnaire Item	Non-Drug Group (N=809)	Psychedelic Group (N=3476)
Details of initiating the encounter (proportion endorsing the item)		
Went into the experience with the intention of encountering that which was encountered	0.16 (0.015)	0.22 (0.007)
The encounter was initiated by that which was encountered (not by me)	0.54 (0.021)	**0.45 (0.009)**
Was alone (not with other people) at the time of the encounter	0.66 (0.020)	**0.33 (0.009)**
Senses with which you interacted during the encounter (proportion endorsing the item)		
Visual	0.49 (0.022)	**0.75 (0.008)**
Auditory (aural)	0.39 (0.020)	**0.48 (0.009)**
Bodily sensation/tactile (sense of touch)	0.46 (0.021)	0.47 (0.009)
Taste (gustatory)	0.03 (0.007)	**0.09 (0.005)**
Smell (olfactory)	0.07 (0.010)	0.11 (0.006)
Extrasensory	0.67 (0.021)	**0.86 (0.006)**
Communication (proportion endorsing the item)		
There was communication (1-way or 2-way exchange of information)	0.62 (0.021)	0.68 (0.008)
Communication was a 2-way exchange of information	0.20 (0.016)	0.25 (0.008)
Communication was a 1-way exchange of information (from it to you)	0.24 (0.018)	0.25 (0.008)
Communication was a 1-way exchange of information (from you to it)	0.04 (0.008)	0.02 (0.002)
Communication was visual (e.g. gestures)	0.17 (0.016)	**0.24 (0.008)**
Communication was verbal-auditory	0.27 (0.019)	0.21 (0.007)
Communication was somatic (e.g. touch/kinesthetic)	0.18 (0.017)	0.13 (0.006)
Communication was extrasensory-telepathic	0.43 (0.021)	**0.60 (0.009)**
Immediate results of the encounter (proportion endorsing the item)		
You had an emotional response during the encounter	0.90 (0.013)	0.89 (0.006)
That which was encountered had an emotional response during the encounter	0.24 (0.018)	0.24 (0.008)
You ascertained a message, task, mission, or insight from the encounter	0.74 (0.019)	0.76 (0.008)
You acquired predictions about the future	0.23 (0.018)	0.24 (0.008)

[1] Within a row, bold font indicates significant difference from the Non-Drug Group.
[2] Data are estimated means (with standard errors of the estimate). Dichotomous data for endorsement and non-endorsement of survey items were coded as 1 and 0 with mean approximating the proportion of the participants in the group that endorsed the item as positive. Statistical comparisons were adjusted for eight covariates (see Statistical section). Results were considered significant when $p \leq 0.001$.

Supporting Information Table B: Details of the encounter in the Non-Drug, Psilocybin, LSD, Ayahuasca, and DMT groups. Data are estimated means and standard errors of the estimate for data from manuscript Table 4[1,2]

Questionnaire Item	Non-Drug Group (N=809)	Psilocybin Group (N=1184)	LSD Group (N=1251)	Ayahuasca Group (N=435)	DMT Group (N=606)
Details of initiating the encounter (proportion endorsing the item)					
Went into the experience with the intention of encountering that which was encountered	0.17 (0.015)	0.20 (0.012)[a]	0.19 (0.011)[a]	**0.27 (0.022)**[a,b]	**0.28 (0.019)**[b]
The encounter was initiated by that which was encountered (not by me)	0.55 (0.021)	**0.44 (0.015)**[b]	**0.37 (0.014)**[b]	0.57 (0.024)[a]	0.55 (0.021)[a]
Was alone (not with other people) at the time of the encounter	0.64 (0.020)	**0.41 (0.015)**[a]	**0.35 (0.014)**[a]	**0.12 (0.016)**[b]	**0.33 (0.020)**[a]
Senses with which you interacted during the encounter (proportion endorsing the item)					
Visual	0.49 (0.022)	**0.72 (0.013)**[a]	**0.74 (0.013)**[a]	**0.74 (0.022)**[a,b]	**0.82 (0.016)**[b]
Auditory (aural)	0.39 (0.020)	0.44 (0.015)[a]	0.48 (0.015)[a,b]	**0.52 (0.025)**[a,b]	**0.56 (0.021)**[b]
Bodily sensation/tactile (sense of touch)	0.46 (0.021)	0.44 (0.015)[a]	0.49 (0.015)[a]	0.49 (0.025)[a]	0.45 (0.021)[a]
Taste (gustatory)	0.03 (0.007)	**0.07 (0.008)**[a]	**0.10 (0.009)**[a]	0.09 (0.015)[a]	**0.10 (0.012)**[a]
Smell (olfactory)	0.07 (0.011)	0.09 (0.009)[a]	0.12 (0.010)[a]	0.13 (0.017)[a]	0.12 (0.013)[a]
Extrasensory	0.67 (0.021)	**0.86 (0.010)**[a]	**0.85 (0.011)**[a]	**0.86 (0.017)**[a]	**0.89 (0.014)**[a]
Communication (proportion endorsing the item)					
There was communication (1-way or 2-way exchange of information)	0.63 (0.021)	0.64 (0.014)[a]	0.60 (0.015)[a]	**0.80 (0.019)**[b]	**0.80 (0.017)**[b]
Communication was a 2-way exchange of information	0.22 (0.017)	0.23 (0.013)[a]	0.20 (0.012)[a]	**0.40 (0.024)**[b]	0.27 (0.019)[a]
Communication was a 1-way exchange of information (from it to you)	0.24 (0.018)	0.23 (0.012)[a]	0.21 (0.012)[a]	0.23 (0.021)[a]	**0.37 (0.020)**[b]
Communication was a 1-way exchange of information (from you to it)	0.04 (0.008)	0.02 (0.004)[a]	0.02 (0.004)[a]	0.02 (0.006)[a]	0.01 (0.004)[a]
Communication was visual (e.g. gestures)	0.17 (0.016)	0.22 (0.012)[a,b]	0.20 (0.012)[a]	**0.30 (0.023)**[b,c]	**0.33 (0.020)**[c]
Communication was verbal-auditory	0.27 (0.019)	0.21 (0.012)[a]	0.19 (0.011)[a]	0.26 (0.022)[a]	0.24 (0.018)[a]
Communication was somatic (e.g. touch/kinesthetic)	0.19 (0.017)	0.11 (0.009)[a]	0.11 (0.009)[a]	0.19 (0.020)[a]	0.17 (0.016)[a]
Communication was extrasensory-telepathic	0.44 (0.021)	**0.57 (0.015)**[a]	0.52 (0.015)[a]	**0.72 (0.022)**[b]	**0.75 (0.018)**[b]

Immediate results of the encounter (proportion endorsing the item)					
You had an emotional response during the encounter	0.90 (0.013)	0.89 (0.009)[a]	0.90 (0.009)[a]	0.87 (0.016)[a]	0.89 (0.013)[a]
That which was encountered had an emotional response during the encounter	0.25 (0.018)	0.22 (0.012)[a]	0.21 (0.012)[a]	0.28 (0.022)[a,b]	0.32 (0.020)[b]
You ascertained a message, task, mission, or insight from the encounter	0.75 (0.018)	0.75 (0.013)[a]	0.74 (0.013)[a]	0.82 (0.019)[a]	0.74 (0.018)[a]
You acquired predictions about the future	0.23 (0.018)	0.23 (0.012)[a]	0.24 (0.013)[a]	0.29 (0.023)[a]	0.19 (0.016)[a]

[1] Within a row, bold font indicates significant difference from the Non-Drug Group; for the drug groups, values not sharing a common letter are significantly different.

[2] Data are estimated means (with standard errors of the estimate). Dichotomous data for endorsement and non-endorsement of survey items were coded as 1 and 0 with mean approximating the proportion of the participants in the group that endorsed the item as positive. Statistical comparisons were adjusted for eight covariates (see Statistical section). Pairwise comparisons were adjusted using Bonferroni method and results were considered significant when p≤0.001.

Supporting Information Table C: Memory, realism, and mystical features of the encounter experience in the Non-Drug Group and combined Psychedelic Group. Data are estimated means and standard errors of the estimate for data from manuscript Table 5[1,2]

Questionnaire Item	Non-Drug Group (N=809)	Psychedelic Group (N=3476)
Memory for encounter (ratings from 0 to 100)		
Vividness of memories of the encounter	85.8 (0.89)	**77.6 (0.38)**
Realism of the encounter (ratings from 0 to 100)		
Superficial dream-like level of reality	28.4 (1.53)	**41.1 (0.65)**
Reality similar to everyday normal consciousness	54.1 (1.52)	**40.7 (0.65)**
More real than everyday normal consciousness	72.0 (1.37)	76.7 (0.58)
Mystical Experience Questionnaire: Factor and total scores (proportion of maximum possible score)		
Mystical factor	0.71 (0.007)	**0.81 (0.003)**
Positive mood factor	0.77 (0.008)	**0.80 (0.003)**
Transcendence of time and space factor	0.54 (0.011)	**0.73 (0.005)**
Ineffability factor	0.77 (0.008)	**0.85 (0.003)**
Total Score	0.70 (0.007)	**0.80 (0.003)**
Mystical Experience Questionnaire: "Complete" mystical experience		
Proportion of group fulfilling criteria for complete experience	0.42 (0.021)	**0.64 (0.009)**

[1] Within a row, bold font indicates significant difference from the Non-Drug Group.

[2] Data are estimated means (with standard errors of the estimate). Dichotomous data for complete and incomplete mystical experiences were coded as 1 and 0 with mean approximating the proportion of the participants in the group that had a complete mystical experience. Statistical comparisons for continuous and dichotomous data were adjusted for eight covariates (see Statistical section). Results were considered significant when p≤0.001.

Supporting Information Table D: Memory, realism, and mystical features of the encounter experience in the Non-Drug, Psilocybin, LSD, Ayahuasca, and DMT groups. Data are estimated means and standard errors of the estimate for data from manuscript Table 6[1,2]

Questionnaire Item	Non-Drug Group (N=809)	Psilocybin Group (N=1184)	LSD Group (N=1251)	Ayahuasca Group (N=435)	DMT Group (N=606)
Memory for the encounter (ratings from 0 to 100)					
Vividness of memories of the encounter	86.0 (0.89)	**77.2 (0.64)**[a]	**78.4 (0.63)**[a]	**79.1 (1.06)**[a]	**75.3 (0.90)**[a]
Realism of the encounter (ratings from 0 to 100)					
Superficial dream-like level of reality	28.0 (1.54)	**41.8 (1.10)**[a]	**39.6 (1.08)**[a]	**38.9 (1.82)**[a]	**45.2 (1.54)**[a]
Reality similar to everyday normal consciousness	54.5 (1.53)	**42.1 (1.09)**[a]	**40.7 (1.08)**[a]	**43.1 (1.82)**[a]	**35.6 (1.54)**[a]
More real than everyday normal consciousness	72.2 (1.38)	74.9 (0.98)[a]	77.0 (0.97)[a]	78.9 (1.63)[a]	77.8 (1.38)[a]
Mystical Experience Questionnaire: factor and total scores (proportion of maximum possible score)					
Mystical factor	0.72 (0.008)	**0.81 (0.005)**[a]	**0.81 (0.005)**[a]	**0.83 (0.009)**[a]	**0.82 (0.008)**[a]
Positive mood factor	0.77 (0.008)	0.79 (0.006)[a]	0.79 (0.006)[a]	0.82 (0.009)[a]	0.81 (0.009)[a]
Transcendence of time and space factor	0.54 (0.010)	**0.70 (0.007)**[a]	**0.70 (0.007)**[a]	**0.73 (0.012)**[a]	**0.84 (0.010)**[b]
Ineffability factor	0.77 (0.008)	**0.84 (0.006)**[a]	**0.84 (0.006)** [a]	**0.85 (0.009)**[a,b]	**0.87 (0.008)**[b]
Total Score	0.70 (0.007)	**0.79 (0.005)**[a]	**0.79 (0.005)**[a]	**0.81 (0.008)**[a,b]	**0.83 (0.007)**[b]
Mystical Experience Questionnaire: "Complete" mystical experience					
Proportion of group fulfilling criteria for complete experience	0.42 (0.021)	**0.63 (0.014)**[a]	**0.61 (0.014)**[a]	**0.66 (0.023)**[a,b]	**0.73 (0.019)**[b]

[1] Within a row, bold font indicates significant difference from the Non-Drug Group; for the drug groups, values not sharing a common letter are significantly different.

[2] Data are estimated means (with standard errors of the estimate). Dichotomous data for complete and incomplete mystical experiences were coded as 1 and 0 with mean approximating the proportion of the participants in the group that had a complete mystical experience. Statistical comparisons for continuous and dichotomous data were adjusted for eight covariates (see Statistical section). For both types of analyses, pairwise comparisons were adjusted using Bonferroni method to control for Type 1 error. Results were considered significant when $p \leq 0.001$.

Supporting Information Table E: Interpretation of that which was encountered in the Non-Drug Group and combined Psychedelic Group. Data are estimated means for dichotomous data from manuscript Table 7[1,2,3]

Questionnaire Item	Non-Drug Group (N=809)	Psychedelic Group (N=3476)
Best descriptor of that which was encountered (proportion endorsing the item)		
God (the God of your understanding)	0.43 (0.022)	**0.17 (0.007)**
Ultimate Realty	0.25 (0.017)	**0.55 (0.009)**
Higher Power	0.16 (0.015)	0.19 (0.007)
An aspect or emissary of God (e.g. an angel)	0.15 (0.015)	**0.09 (0.005)**
Attributes to that which was encountered (proportion endorsing the item)[3]		
Benevolent (i.e. kind, compassionate, altruistic)	0.83 (0.016)	**0.72 (0.008)**
Intelligent	0.78 (0.018)	0.79 (0.007)
Sacred	0.77 (0.018)	0.72 (0.008)
Conscious (i.e. self-aware)	0.68 (0.020)	0.69 (0.008)
Eternal	0.67 (0.020)	0.71 (0.008)
All Knowing	0.64 (0.020)	0.60 (0.009)
Agency (e.g. could it affect outcomes, events, or material objects in this reality)	0.48 (0.021)	**0.35 (0.008)**
Petitionable (e.g. in response to prayer or petition, it might change events or circumstances)	0.33 (0.021)	**0.17 (0.007)**
Positively Judgmental (e.g. inclined toward strong approval or reward)	0.26 (0.019)	0.27 (0.008)
Negatively Judgmental (e.g. inclined toward strong disapproval or harsh punishment)	0.06 (0.010)	0.07 (0.005)
Malicious (i.e., unkind, cruel, vengeful)	0.02 (0.006)	**0.08 (0.005)**
Additional interpretation of that which was encountered (proportion endorsing the item)		
That which was encountered existed, as least in part, in some other dimension or reality	0.65 (0.020)	0.70 (0.008)
You were completely the same as that which was encountered	0.31 (0.019)	**0.47 (0.009)**
That which was encountered continued to exist after the encounter	0.69 (0.020)	0.66 (0.008)

[1] Within a row, bold font indicates significant difference from the Non-Drug Group.
[2] Data are estimated means (with standard errors of the estimate). Dichotomous data for endorsement and non-endorsement of survey items were coded as 1 and 0 with the mean approximating the proportion of the participants in the group that endorsed the item as positive. Statistical comparisons were adjusted for eight covariates (see Statistical section). Results were considered significant when $p \leq 0.001$.
[3] Response options for these questions were Yes, No, and I don't know.

Supporting Information Table F: Interpretation of that which was encountered in Non-Drug, Psilocybin, LSD, Ayahuasca, and DMT groups. Data are estimated means for dichotomous data from manuscript Table 8[1,2,3]

Items	Non-Drug Group (N=809)	Psilocybin Group (N=1184)	LSD Group (N=1251)	Ayahuasca Group (N=435)	DMT Group (N=606)
Best descriptor of that which was encountered (proportion endorsing the item)					
God (the God of your understanding)	0.44 (0.023)	**0.15 (0.011)**[a]	**0.18 (0.011)**[a]	**0.22 (0.021)**[a]	**0.15 (0.015)**[a]
Ultimate Realty	0.25 (0.017)	**0.57 (0.015)**[a,b]	**0.59 (0.014)**[b]	**0.46 (0.025)**[a]	**0.48 (0.021)**[a]
Higher Power	0.16 (0.016)	0.18 (0.011)[a,b]	0.16 (0.011)[a]	0.20 (0.020)[a,b]	0.24 (0.018)[b]
An aspect or emissary of God (e.g. an angel)	0.15 (0.015)	0.09 (0.009)[a]	**0.06 (0.007)**[a]	0.11 (0.015)[a]	0.12 (0.014)[a]
Attributes to that which was encountered (proportion endorsing the item)[3]					
Benevolent (i.e. kind, compassionate, altruistic)	0.83 (0.016)	**0.68 (0.014)**[b]	**0.68 (0.014)**[b]	0.86 (0.017)[a]	0.77 (0.017)[a]
Intelligent	0.79 (0.017)	0.74 (0.013)[a]	0.74 (0.013)[a]	**0.91(0.014)**[b]	**0.88 (0.013)**[b]
Sacred	0.78 (0.018)	0.73 (0.013)[a,b]	0.69 (0.014)[b]	0.81 (0.019)[a]	0.71 (0.019)[a,b]
Conscious (i.e. self-aware)	0.69 (0.020)	0.63 (0.014)[a]	0.65 (0.014)[a]	**0.80 (0.020)**[b]	0.78 (0.017)[b]
Eternal	0.68 (0.020)	0.70 (0.014)[a]	0.73 (0.013)[a]	0.74 (0.022)[a]	0.64 (0.020)[a]
All Knowing	0.65 (0.020)	0.58 (0.015)[a]	0.58 (0.015)[a]	0.66 (0.023)[a]	0.59 (0.021)[a]
Agency (e.g. could it affect outcomes, events, or material objects in this reality)	0.49 (0.021)	**0.33 (0.014)**[a]	**0.37 (0.014)**[a]	0.42 (0.024)[a]	**0.30 (0.019)**[a]
Petitionable (e.g. in response to prayer or petition, it might change events or circumstances)	0.35 (0.021)	**0.16 (0.011)**[b]	**0.16 (0.011)**[b]	0.28 (0.023)[a]	**0.14 (0.014)**[b]
Positively Judgmental (e.g. inclined toward strong approval or reward)	0.27 (0.019)	0.27 (0.013)[a]	0.24 (0.013)[a]	0.32 (0.024)[a]	0.30 (0.019)[a]
Negatively Judgmental (e.g. inclined toward strong disapproval or harsh punishment)	0.06 (0.010)	0.07 (0.007)[a]	0.07 (0.007)[a]	0.07 (0.013)[a]	0.09 (0.012)[a]
Malicious (i.e., unkind, cruel, vengeful)	0.02 (0.006)	**0.08 (0.008)**[a]	**0.09 (0.008)**[a]	**0.08 (0.013)**[a]	**0.07 (0.010)**[a]
Additional interpretation of that which was encountered (proportion endorsing the item)					
That which was encountered existed, as least in part, in some other dimension or reality	0.65 (0.020)	0.66 (0.014)[a]	0.67 (0.014)[a,b]	**0.77 (0.021)**[b,c]	**0.76 (0.018)**[c]
You were completely the same as that which was encountered	0.31 (0.019)	**0.47 (0.015)**[a,b]	**0.53 (0.015)**[b]	**0.43 (0.024)**[a,b]	0.42 (0.021)[a]

That which was encountered continued to exist after the encounter	0.70 (0.020)	0.65 (0.014)[a]	0.69 (0.014)[a]	0.75 (0.022)[a]	**0.55 (0.021)**[b]

[1] Within a row, bold font indicates significant difference from the Non-Drug Group; for the drug groups, values not sharing a common letter are significantly different

[2] Data are estimated means (with standard errors of the estimate). Dichotomous data for endorsement and non-endorsement of survey items were coded as 1 and 0 with the mean approximating the proportion of the participants in the group that endorsed the item as positive. Statistical comparisons were adjusted for eight covariates (see Statistical section). Pairwise comparisons were adjusted using Bonferroni method and results were considered significant when $p \leq 0.001$.

[3] Response options for these questions were Yes, No, and I don't know.

Supporting Information Table G: Comparison of encounter experience relative to other lifetime experiences in the Non-Drug Group and combined Psychedelic Group. Data are estimated means and standard errors of the estimate for data from manuscript Table 9[1,2,3]

Questionnaire Item	Non-Drug Group (N=809)	Psychedelic Group (N=3476)
Rating relative to other lifetime experiences (ratings from 1 to 8)		
How personally meaningful was the encounter	6.85 (0.044)	6.91 (0.019)
How spiritually significant was the encounter	6.94 (0.054)	7.04 (0.023)
How personally psychologically insightful was the encounter	6.03 (0.071)	**6.51 (0.031)**
How psychologically challenging was the encounter	4.54 (0.102)	**5.19 (0.044)**
Proportion rating the item as among the top 5 or single most of lifetime		
How personally meaningful was the encounter	0.75 (0.018)	0.78 (0.007)
How spiritually significant was the encounter	0.79 (0.017)	0.83 (0.007)
How personally psychologically insight was the encounter	0.61 (0.020)	0.67 (0.008)
How psychologically challenging was the encounter	0.38 (0.020)	0.43 (0.009)
Proportion rating the item as the single most of lifetime		
How personally meaningful was the encounter	0.31 (0.020)	0.26 (0.008)
How spiritually significant was the encounter	0.39 (0.021)	0.41 (0.009)
How personally psychologically insight was the encounter	0.24 (0.018)	0.27 (0.008)
How psychologically challenging was the encounter	0.15 (0.015)	0.16 (0.007)

[1] Within a row, bold font indicates significant difference from the Non-Drug Group.

[2] Data are estimated means (with standard errors of the estimate). Dichotomous data for endorsement and non-endorsement of survey items were coded as 1 and 0 with mean approximating the proportion of the participants in the group that endorsed the items as positive. Statistical comparisons for continuous and dichotomous data were adjusted for eight covariates (see Statistical section). Results were considered significant when $p \leq 0.001$.

[3] Rating options ranged from 1=no more than routine, everyday experience; 5=similar to experiences that occur on average once every 5 years; 6=among the 10 most in my life; 7=among the 5 most of my life; 8=the single most of my life.

Supporting Information Table H: Comparison of encounter experience relative to other lifetime experiences in the Non-Drug, Psilocybin, LSD, Ayahuasca, and DMT groups. Data are estimated means standard errors of the estimate for data from manuscript Table 10[1,2,3]

Questionnaire Item	Non-Drug Group (N=809)	Psilocybin Group (N=1184)	LSD Group (N=1251)	Ayahuasca Group (N=435)	DMT Group (N=606)
Rating relative to other lifetime experiences (ratings from 1 to 8)					
How personally meaningful was the encounter	6.87 (0.045)	6.83 (0.032)[a]	6.88 (0.031)[a,b]	7.12 (0.053)[b]	6.95 (0.045)[a,b]
How spiritually significant was the encounter	6.96 (0.054)	6.98 (0.039)[a]	6.96 (0.038)[a]	**7.32 (0.064)**[b]	7.10 (0.054)[a,b]
How personally psychologically insightful was the	6.06 (0.072)	**6.46 (0.051)**[a]	**6.46 (0.051)**[a]	**6.81 (0.085)**[a]	**6.47 (0.072)**[a]
How psychologically challenging was the encounter	4.58 (0.103)	5.03 (0.073)[a]	**5.16 (0.073)**[a,b]	**5.67 (0.122)**[b]	**5.19 (0.103)**[a,b]
Proportion rating the item as among the top 5 or single most of lifetime					
How personally meaningful was the encounter	0.75 (0.018)	0.75 (0.013)[a]	0.77 (0.012)[a,b]	0.84 (0.018)[b]	0.80 (0.017)[a,b]
How spiritually significant was the encounter	0.80 (0.017)	0.81 (0.012)[a]	0.81 (0.012)[a]	**0.89 (0.015)**[b]	0.84 (0.016)[a,b]
How personally psychologically insight was the encounter	0.62 (0.020)	0.64 (0.014)[a]	0.67 (0.014)[a]	0.72 (0.022)[a]	0.66 (0.020)[a]
How psychologically challenging was the encounter	0.38 (0.021)	0.40 (0.015)[a]	0.42 (0.015)[a]	0.48 (0.025)[a]	0.44 (0.021)[a]
Proportion rating the item as the single most of lifetime					
How personally meaningful was the encounter	0.25 (0.018)	**0.23 (0.012)**[a]	0.27 (0.013)[a]	0.35 (0.024)[b]	0.26 (0.018)[a,b]
How spiritually significant was the encounter	0.40 (0.021)	0.39 (0.015)[a]	0.39 (0.014)[a]	0.49 (0.025)[a]	0.43 (0.021)[a]
How personally psychologically insight was the encounter	0.25 (0.018)	0.23 (0.012)[a]	0.27 (0.013)[a,b]	0.35 (0.024)[b]	0.26 (0.018)[a,b]
How psychologically challenging was the encounter	0.15 (0.016)	0.13 (0.010)[a]	0.16 (0.011)[a,b]	0.22 (0.021)[b]	0.16 (0.015)[a,b]

[1] Within a row, bold font indicates significant difference from the Non-Drug Group; for the drug groups, values not sharing a common letter are significantly different.

[2] Data are estimated means (with standard errors of the estimate). Dichotomous data for endorsement and non-endorsement of survey items were coded as 1 and 0 with mean approximating the proportion of the participants in the groups that endorsed the item as positive. Statistical comparisons for continuous and dichotomous data were adjusted for eight covariates (see Statistical section). For both types of analyses, pairwise comparisons were adjusted using Bonferroni method to control for Type 1 error. Results were considered significant when p≤0.001.

[3] Rating options ranged from 1=no more than routine, everyday experience; 5=similar to experiences that occur on average once every 5 years; 6=among the 10 most in my life; 7=among the 5 most of my life; 8=the single most of my life.

Supporting Information Table I: Persisting changes attributed to the encounter in the Non-Drug Group and combined Psychedelic Group. Data are estimated means and standard errors of the mean for data from manuscript Table 11[1,2]

Questionnaire Item	Non-Drug Group (N=809)	Psychedelic Group (N=3476)
Persisting changes attributed to the encounter experience (ratings from -3 to +3)[3]		
Personal sense of well-being or life satisfaction	2.26 (0.042)	2.24 (0.018)
Your life's purpose	2.11 (0.047)	1.98 (0.020)
Your life's meaning	2.13 (0.047)	2.01 (0.020)
Your social relationships (e.g. family, friends, neighbors, strangers etc.)	1.71 (0.052)	1.69 (0.022)
Your spiritual awareness in everyday life	2.38 (0.040)	**2.17 (0.017)**
Your attitudes about life	2.21 (0.041)	2.20 (0.018)
Your attitudes about self	2.11 (0.044)	2.08 (0.019)
Your mood	1.48 (0.049)	1.54 (0.021)
Your behavior	1.70 (0.047)	1.60 (0.020)
	2.26 (0.042)	2.24 (0.018)
Persisting changes attributed to the encounter experience (proportion endorsing the item)		
Desirable change in contemplative, prayer, or meditation practice	0.89 (0.013)	0.85 (0.006)
Undesirable change in contemplative, prayer, or meditation practice	0.01 (0.005)	0.01 (0.002)
Desirable change in understanding religious or spiritual traditions other than your own	0.83 (0.015)	0.85 (0.006)
Undesirable change in understanding religious or spiritual traditions other than your own	0.02 (0.006)	0.01 (0.002)
Decreased fear of death	0.61 (0.020)	**0.70 (0.008)**
Increased fear of death	0.01 (0.005)	0.03 (0.003)

[1] Within a row, bold font indicates significant difference from the Non-Drug Group.

[2] Data are estimated means (with standard errors of the estimate). Dichotomous data for endorsement and non-endorsement of survey items were coded as 1 and 0 with mean approximating the proportion of the participants in the group that endorsed the item as positive. Statistical comparisons for continuous and dichotomous data were adjusted for eight covariates (see Statistical section). Results were considered significant when $p \leq 0.001$.

[3] Rating options ranged from -3=Strong negative change that I consider undesirable to +2 Moderate positive change that I consider desirable and +3= Strong positive change that I consider desirable.

Supporting Information Table J: Persisting changes attributed to the encounter in the Non-Drug, Psilocybin, LSD, Ayahuasca, and DMT groups. Data are estimated means and standard errors of the estimate for data from manuscript Table 12[1,2]

Items	Non-Drug Group (N=809)	Psilocybin Group (N=1184)	LSD Group (N=1251)	Ayahuasca Group (N=435)	DMT Group (N=606)
Persisting changes attributed to the encounter experience (ratings from -3 to +3)[3]					
Personal sense of well-being or life satisfaction	2.28 (0.042)	2.20 (0.030)[a]	2.17 (0.030)[a]	2.44 (0.050)[b]	2.30 (0.042)[a,b]
Your life's purpose	2.12 (0.047)	1.95 (0.033)[a]	1.93 (0.033)[a]	2.16 (0.055)[a]	2.00 (0.047)[a]
Your life's meaning	2.15 (0.047)	2.01 (0.034)[a,b]	**1.91 (0.033)**[b]	2.21 (0.056)[a]	2.05 (0.047)[a,b]
Your social relationships (e.g. family, friends, neighbors, strangers etc.)	1.75 (0.052)	1.68 (0.037)[a]	1.55 (0.037)[a]	2.04 (0.062)[b]	1.68 (0.052)[a]
Your spiritual awareness in everyday life	2.40 (0.040)	**2.16 (0.028)**[b]	**2.08 (0.028)**[b]	2.38 (0.047)[a]	2.22 (0.040)[a,b]
Your attitudes about life	2.22 (0.041)	2.15 (0.030)[a]	2.14 (0.029)[a]	2.37 (0.049)[b]	2.25 (0.042)[a,b]
Your attitudes about self	2.13 (0.044)	2.04 (0.031)[a]	2.01 (0.031)[a]	2.30 (0.052)[b]	2.10 (0.044)[a,b]
Your mood	1.51 (0.049)	1.52 (0.035)[a]	1.41 (0.035)[a]	**1.82 (0.059)**[b]	1.62 (0.050)[a,b]
Your behavior	1.72 (0.047)	1.57 (0.034)[b]	**1.48 (0.033)**[b]	1.90 (0.056)[a]	1.65 (0.048)[a,b]
Persisting changes attributed to the encounter experience (proportion endorsing the item)					
Desirable change in contemplative, prayer, or meditation practice	0.89 (0.013)	0.86 (0.010)[a]	0.84 (0.011)[a]	0.88 (0.016)[a]	0.85 (0.015)[a]
Undesirable change in contemplative, prayer, or meditation practice	0.01 (0.005)	0.01 (0.003)	0.01 (0.003)	0.01 (0.006)	0.01 (0.004)
Desirable change in understanding religious or spiritual traditions other than your own	0.83 (0.015)	0.84 (0.011)[a]	0.85 (0.011)[a]	0.88 (0.015)[a]	0.85 (0.015)[a]
Undesirable change in understanding religious or spiritual traditions other than your own	0.02 (0.006)	0.02 (0.004)	0.01 (0.003)	0.01 (0.005)	0.02 (0.005)
Decreased fear of death	0.61 (0.021)	0.69 (0.014)[a,b]	0.66 (0.014)[a]	**0.74 (0.021)**[a,b]	**0.76 (0.018)**[b]
Increased fear of death	0.01 (0.005)	0.03 (0.005)[a]	0.03 (0.005)[a]	0.03 (0.009)[a]	0.02 (0.006)[a]

[1] Within a row, bold font indicates significant difference from the Non-Drug Group; for the drug groups, values not sharing a common letter are significantly different.

[2] Data are estimated means (with standard errors of the estimate). Dichotomous data for endorsement and non-endorsement of survey items were

coded as 1 and 0 with the mean approximating the proportion of the participants in the group that endorsed the item as positive. Statistical comparisons for continuous and dichotomous data were adjusted for eight covariates (see Statistical section). For both types of analyses, pairwise comparisons were adjusted using Bonferroni method to control for Type 1 error. Results were considered significant when $p \leq 0.001$.

[3] Rating options ranged from -3=Strong negative change that I consider undesirable to +2 Moderate positive change that I consider desirable and +3= Strong positive change that I consider desirable.
