# Supplementary material for: Survey of subjective "God encounter experiences": Comparisons among naturally occurring experiences and those occasioned by the classic psychedelics psilocybin, LSD, ayahuasca, or DMT
Source: PLoS One. 2019 Apr 23;14(4):e0214377. doi: 10.1371/journal.pone.0214377 (PMC6478303; doi:10.1371/journal.pone.0214377)
Supplement: S3 File — (PDF) [file pone.0214377.s003.pdf]

Supporting Information

S3 Questionnaire of God encounter experiences occasioned by classic psychedelics

From: Griffiths, R.R., Hurwitz, E.S., Davis, A.K., Johnson, M.W., & Jesse, R. Survey of subjective "God encounter experiences": Comparisons among naturally occurring experiences and those occasioned by the classic psychedelics psilocybin, LSD, ayahuasca, or DMT. PLOS One

The questionnaire was presented to participants on a widely used online survey administration website (www.qualtrics.com). The following document, which was downloaded from the Qualtrics website, provides all possible questions and text that could have been presented to a participant. The questionnaire involved presentation logic that skipped some questions and text depending on a given participant's responses. Additionally, in the following document there are instances in which presentation code (e.g. ${q://QID149/ChoiceGroup/SelectedChoices} appears which was replaced by the text of a response that the participant made to a previous question.

Demo Info

Welcome

This is a questionnaire study of personal encounters with the Divine that occur after taking a classic hallucinogen such as psilocybin or LSD. This research study is being conducted by scientists at Johns Hopkins University School of Medicine and has been approved by the Johns Hopkins University Institutional Review Board (IRB). The IRB application number for this research study is NA_00054696 Dr. Roland Griffiths, Ph.D. is the Principal Investigator for this research study. Detailed information about the study is provided in the next few pages. If you need further information please contact ***EncounteringTheDivine@jhu.edu***

Our Previous Research
Our research team has previously conducted studies investigating spirituality, religion, mystical experiences, and altered states of consciousness occasioned by psychedelic drugs.

Purpose of This Study
In this survey, we want to characterize various experiences of encounters with something that someone might call: "God" (e.g., the God of your understanding), "Higher Power," "Ultimate Reality," or an Aspect or Emissary of God (e.g., an angel).

Eligibility Criteria
You are invited to participate in this survey if you fulfill all of the criteria listed below.
1) You are at least 18 years old.
2) You read, write, and speak English fluently.
3) You have taken a dose of a classic hallucinogen (e.g., psilocybin mushrooms, psilocybin, LSD, ayahuasca, mescaline, DMT, etc.) that produced moderate to strong psychoactive effects.
4) After taking the substance you have had an experience of encountering something that someone might call: "God" (e.g., the God of your understanding), "Higher Power," "Ultimate Reality," or an Aspect or Emissary of God (e.g., an angel).

What the Study Entails
Participation in this study involves filling out an online survey that will take approximately **45-60 minutes**. **You will be required to complete the survey in one sitting.**

Why should I participate?
We believe that this study is scientifically important and may be personally worthwhile to you. We would like you to participate because we need to collect responses from many different people in order to better characterize experiences of such encounters. You may find this survey interesting and an uplifting opportunity to revisit and contemplate a precious experience. You may also find it meaningful to further explore some of your philosophical and theological beliefs. However, you may find it to be uncomfortable because it will prompt you to explain some of these deeply held beliefs. There is also a chance that you will be bored. Although there is no monetary compensation for participation, you will be making a unique and important scientific contribution, and you may gain further insights into your experiences.

Is my participation confidential?
Yes. We do not collect identifying information such as your name, email address, or IP address. Your anonymous responses will be seen and analyzed by Johns Hopkins staff or representatives. To further protect the confidentiality

of participants, the results of most questions will be presented in aggregate. If you complete the optional openended section of the survey, we may quote from your textual responses. However, if you provide specific identifying information, we will edit your responses to protect your confidentiality.

What kind of information will I be providing?
At the beginning of the survey, you will be asked questions about your background, spiritual, and religious beliefs. Most of the survey asks about your experience(s) during an encounter with something that someone might call: "God" (e.g., the God of your understanding), "Higher Power," "Ultimate Reality," or an Aspect or Emissary of God (e.g., an angel). At the end of the survey, there will be an optional open ended section that could add 10-15 minutes to the total duration. You will not be required to complete the open ended section.

What will become of the results from this study?
The researchers intend to publish the results from this study in the scientific literature and to present results at scientific meetings. We will also make the results publicly available by posting a notice of any scholarly publications on the website of the Council on Spiritual Practices (www.csp.org).

Your responses will not be used in this study if you do not complete the survey.
Your participation in this study is voluntary. Your completion of this survey or questionnaire will serve as your consent to be in this research study. Even after you begin the survey, you may stop answering the questions at any time. If you stop early, none of your responses will be used.

How do I start?
You can begin the survey by clicking 'Next' at the bottom of this page. This survey will take approximately **45-60 minutes** and **you will be required to complete the survey in one sitting.**
It is important that you complete the survey only once, and that you answer each question honestly and seriously. If you are not ready to complete the survey now, please return to this page at a time that is convenient for you.
You can exit the survey at any time by clicking the link in the upper right corner of your screen. If you exit the survey early, your responses will not be used.

During the survey, **please do not press the "back" button on your internet browser** as it may erase your answers or prematurely terminate your session.

By clicking 'Next' below, you affirm that

• you have read the information above
• you fulfill the eligibility criteria
• you voluntarily agree to participate
• you are at least 18 years old

[**Technical note**: Certain browser plugins or extensions could cause display or functionality problems with the survey. If you run into any technical difficulties, please try to disable your plugins and reload the browser. For further assistance in the event of such difficulties, please email us at EncounteringTheDivine@jhu.edu]

Click below to begin or exit the survey:

Begin survey	Exit survey
○	○

Have you completed this survey before?

Yes	No
○	○

Are you 18 years or older?

Yes	No
○	○

Do you read, write, and speak English fluently?

Yes	No
○	○

Have you ever taken a dose of a classic hallucinogen (e.g., psilocybin mushrooms, psilocybin, LSD, ayahuasca, mescaline, DMT, etc.) that produced moderate to strong psychoactive effects?

Yes	No
○	○

After taking the substance, did you have an experience of encountering something that someone might call: "God" (e.g., the God of your understanding), "Higher Power," "Ultimate Reality," or an Aspect or Emissary of God (e.g., an angel)?

Yes	No
○	○

We sincerely appreciate your interest in this survey and your willingness to volunteer your time. Unfortunately, the inclusion criteria for this survey are restrictive and you do not meet all of the criteria needed to participate. In the future, we hope to initiate another version of this survey with less restrictive inclusion requirements. We hope that you may be interested in participating in such a survey in the future.

With gratitude for your interest,

The JHU Spirituality Research Team

Note: none of the responses you've already given will be used in the study.

What year were you born?

What is your sex?

- Male
- Female

What is your race?

- Caucasian / White
- African American / Black
- Asian
- Native Hawaiian or Pacific Islander
- Native American
- Mixed Race

Are you Hispanic or Latino? ('Hispanic' or 'Latino' refers to a person of Cuban, Mexican, Puerto Rican, South or Central American, or other Spanish culture or origin, regardless of race)

Yes	No
○	○

In which country do you reside?

What is your total annual household income (before taxes)?

- Under $25,000
- $25,000-$34,999
- $35,000-$49,999
- $50,000-$74,999
- $75,000-$99,999
- $100,000-$124,999
- $125,000-$150,000
- Over $150,000

What is your level of education?

- No High School diploma
- High school Diploma or equivalent (GED)
- Some college credit, no degree
- Trade, Technical, Vocational training after high school
- Associate's degree
- Bachelor's Degree
- Master's Degree
- Advanced professional or Doctoral Degree (e.g., Ph.D., M.D., etc.)

What is your marital or partnership status?

- Married
- Living with partner
- Divorced or separated
- Widowed
- Never married

Have you ever been diagnosed by a medical professional as having a psychotic disorder (e.g. schizophrenia or another psychiatric disorder characterized by delusions or hallucinations)?

Yes	No
◯	◯

Which of the following would best characterize your **current** religious orientation?

- Agnostic
- Atheist
- Falun Gong
- Hindu
- Native American
- Nature-based
- Secular Humanist
- Shinto

◯ Baha'i	◯ Islam	◯ Pagan	◯ Taosim
◯ Buddhist	◯ Jainism	◯ Pantheist (belief in the divinity of the universe)	◯ Unitarian Universalist
◯ Christian	◯ Jewish	◯ Scientology	◯ Zoroastrian
◯ Earth-based	◯ Nation of Islam	◯ Secular (no religious or spiritual orientation)	◯ Other

Please describe (50 characters or less):

Please specify:

◯ Catholic	◯ Methodist	◯ Calvanist
◯ Eastern Orthodox	◯ Quaker	◯ Adventist
◯ Episcopalian	◯ Pentecostal	◯ Congregationalist
◯ Baptist	◯ Nondenomenational	◯ Mormon
◯ Lutheran	◯ Anglican	◯ Other
◯ Presbyterian		

Please specify:

◯ Sunni
◯ Shi'ah
◯ Khawarij
◯ Sufi
◯ Other

Please specify:

- Orthodox
- Conservative
- Reform
- Reconstructionist
- Other

To what extent do you consider yourself a religious person?

- Very religious
- Moderately religious
- Slightly religious
- Not religious at all

To what extent do you consider yourself a spiritual person?

- Very spiritual
- Moderately spiritual
- Slightly spiritual
- Not spiritual at all

Choose one statement that best defines your religiousness and spirituality, as you interpret the meaning of these terms:

- I am spiritual and religious
- I am spiritual but not religious
- I am religious but not spiritual
- I am neither spiritual nor religious

About how often do you spend time on religious or spiritual practices?

Several times per day	Once per day	Several times per week	Once per week	Several times per month	Once per month	Several times per year	Once a year or less

God

Previously, you indicated that you have had an experience of encountering something that someone might call: "God" (e.g., the God of your understanding), "Higher Power," "Ultimate Reality," or an Aspect or Emissary of God (e.g., an angel) after taking a classic hallucinogen (e.g., psilocybin mushrooms, psilocybin, LSD, ayahuasca, mescaline, DMT, etc.).

Take a few moments now to recollect and bring to mind that particular experience. If you have had multiple such experiences, please answer the following with respect to the **single most memorable experience**. Please answer the following questions according to your feelings and thoughts about that experience. We understand that response choices may not exactly match your experience, so please select the best of the provided responses. At the end of this survey, you will have an optional opportunity to further describe your experiences or to write clarifying notes about any of your answers.

Which of the following best describes what you encountered?

Note: Your answer will be used as the descriptor for subsequent questions throughout this survey. ***Again, we realize that this may not be the BEST descriptor for what you encountered. However, we need a descriptor to be used as a referent in subsequent questions throughout the survey.***

God (e.g., the God of your understanding)	Higher Power	Ultimate Reality	An Aspect or Emissary of God (e.g., an angel)
◯	◯	◯	◯

Please describe briefly what you encountered (1000 characters or less):

What classic hallucinogen do you believe you had taken when the encounter occurred?

◯ Psilocybin mushrooms

◯ 5-MeO-DMT

- Psilocybin
- LSD (Acid)
- Ayahuasca
- DMT (other than Ayahuasca)
- Mescaline
- Peyote cactus
- Other

If other, please describe briefly which classic hallucinogen (200 characters or less):

What dose did you take before your encounter?

Note: Please be as descriptive as possible to the best of your knowledge (e.g., 8 g dried Psilocybe cubensis mushrooms, 100 micrograms LSD, 4 tabs of LSD, etc.)

How would you characterize this dose?

1 Low	2 Moderate	3 Moderately High	4 High
○	○	○	○

Approximately how old were you when you had this experience?

Did you go into this experience with the intention of trying to encounter the previously identified ${q://QID149/ChoiceGroup/SelectedChoices}?

Yes	No
○	○

Have you ever encountered the previously identified ${q://QID149/ChoiceGroup/SelectedChoices} before?

Yes	No	I do not know
○	○	○

Some people have experiences of an awareness or awakening to ${q://QID149/ChoiceGroup/SelectedChoices} in which they experience absolutely no differentiation between themselves and ${q://QID149/ChoiceGroup/SelectedChoices}. Thinking about the most salient portion of the encounter, rate the degree to which you felt that you and ${q://QID149/ChoiceGroup/SelectedChoices} were the completely different vs completely the same.

Completely different	Very different	Moderately different	Slightly different	Completely the same (Not at all different)
○	○	○	○	○

With what senses did you interact with the previously identified ${q://QID149/ChoiceGroup/SelectedChoices}?

[Check all that apply]

Visual	Aural (Auditory)	Bodily Sensation/Tactile (Sense of touch)	Gustatory (Taste)	Olfactory (Smell)	Extrasensory
☐	☐	☐	☐	☐	☐

How was the encounter with the previously identified ${q://QID149/ChoiceGroup/SelectedChoices} initiated?

- ○ The previously identified ${q://QID149/ChoiceGroup/SelectedChoices} initiated the encounter
- ○ I initiated the encounter
- ○ Other

During your encounter, was there any communication (e.g., a one-way or two-way exchange of information) between you and the previously identified ${q://QID149/ChoiceGroup/SelectedChoices}?

Yes	No

How did you or the previously identified ${q://QID149/ChoiceGroup/SelectedChoices} communicate? [Check all that apply]

Verbal-Auditory	Visual (e.g., Gestures)	Somatic (e.g., Touch/Kinesthetic)	Extrasensory-Telepathy
☐	☐	☐	☐

What was the style of communication (e.g., a one-way or two-way exchange of information)?

- Question and Answer
- Dialogue (two-way exchange of information)
- Monologue (one-way exchange of information) from the previously identified ${q://QID149/ChoiceGroup/SelectedChoices} to you
- Monologue (one-way exchange of information) from you to the previously identified ${q://QID149/ChoiceGroup/SelectedChoices}
- Other

Did you have an emotional response to your encounter with the previously identified ${q://QID149/ChoiceGroup/SelectedChoices} (this could include expressed or unexpressed emotional responses)?

Yes	No	I do not know
○	○	○

Did the previously identified ${q://QID149/ChoiceGroup/SelectedChoices} have an emotional response to its encounter with you?

Yes	No	I do not know
○	○	○

How novel was the previously identified ${q://QID149/ChoiceGroup/SelectedChoices}? For example, if you visually perceived the previously identified ${q://QID149/ChoiceGroup/SelectedChoices}, have you ever seen or imagined images similar to its appearance? Or if you heard the previously identified ${q://QID149/ChoiceGroup/SelectedChoices}, have you ever heard or imaged noises similar to those you heard?

Completely Familiar | Completely Novel

Did you ascertain a message, task, mission, or insight from your encounter with the previously identified ${q://QID149/ChoiceGroup/SelectedChoices} (this could have been directly imparted to you or attained through your understanding of its nature, etc.)?

Yes No

If yes, please explain (200 characters or less):

Did you acquire any predictions about the future through your encounter with the previously identified ${q://QID149/ChoiceGroup/SelectedChoices}?

Yes No

Thinking back on the encounter, where do you think the previously identified ${q://QID149/ChoiceGroup/SelectedChoices} existed?

- In this dimension or reality
- In some other dimension or reality
- Both in this dimension or reality and some other dimension or reality
- I do not know

Please explain briefly (200 characters or less):

Did the previously identified ${q://QID149/ChoiceGroup/SelectedChoices} continue to exist after your encounter?

- Yes
- No
- I do not know

Did the previously identified ${q://QID149/ChoiceGroup/SelectedChoices} to any extent have agency in the world (e.g. could it affect outcomes, events, or material objects in this reality)?

Yes	No	I do not know
○	○	○

Rate the degree to which the previously identified ${q://QID149/ChoiceGroup/SelectedChoices} had agency in the world:

No Agency — Complete Agency

Did you regard the previously identified ${q://QID149/ChoiceGroup/SelectedChoices} to any extent as sacred?

Yes No I do not know

Rate the degree to which you regard the previously identified ${q://QID149/ChoiceGroup/SelectedChoices} as sacred:

Not at all Sacred — Completely Sacred

Was the previously identified ${q://QID149/ChoiceGroup/SelectedChoices} to any extent intelligent?

Yes | No | I do not know

Rate the degree to which the previously identified ${q://QID149/ChoiceGroup/SelectedChoices} was intelligent:

Not at all Intelligent — Highly Intelligent

Was the previously identified ${q://QID149/ChoiceGroup/SelectedChoices} all-knowing?

Yes | No | I do not know

Was the previously identified ${q://QID149/ChoiceGroup/SelectedChoices} to any extent benevolent (i.e., kind, compassionate, altruistic)?

Yes | No | I do not know

Rate the degree to which the previously identified ${q://QID149/ChoiceGroup/SelectedChoices} was benevolent (i.e., kind, compassionate, altruistic):

Not at all Benevolent | Completely Benevolent

Was the previously identified ${q://QID149/ChoiceGroup/SelectedChoices} to any extent malicious (i.e., unkind, cruel, vengeful)?

Yes | No | I do not know

Rate the degree to which the previously identified ${q://QID149/ChoiceGroup/SelectedChoices} was malicious (i.e., unkind, cruel, vengeful):

Not at all Malicious | Completely Malicious

Was the previously identified ${q://QID149/ChoiceGroup/SelectedChoices} eternal?

Yes | No | I do not know

Was the previously identified ${q://QID149/ChoiceGroup/SelectedChoices} to any extent conscious (i.e., self aware)?

Yes No I do not know

Rate the degree to which the previously identified ${q://QID149/ChoiceGroup/SelectedChoices} was conscious (i.e., self aware):

Not at all Conscious

Completely Conscious

Was the previously identified ${q://QID149/ChoiceGroup/SelectedChoices} to any extent negatively judgmental (e.g. inclined toward strong disapproval or harsh punishment)?

Yes No I do not know

Rate the degree to which the previously identified ${q://QID149/ChoiceGroup/SelectedChoices} was negatively judgmental:

Not at all Negatively Judgmental

Highly Negatively Judgmental

Was the previously identified ${q://QID149/ChoiceGroup/SelectedChoices} to any extent positively judgmental (e.g. inclined toward strong approval or reward)?

[Note: It is possible to endorse both the previous question and this question without contradiction]

Yes No I do not know

Rate the degree to which the previously identified ${q://QID149/ChoiceGroup/SelectedChoices} was positively judgmental:

Not at all Positively Judgmental	Highly Positively Judgmental

Was the previously identified ${q://QID149/ChoiceGroup/SelectedChoices} to any extent petition-able (e.g. in response to prayer or petition, it might change events or circumstances)?

Yes	No	I do not know
○	○	○

Rate the degree to which the previously identified ${q://QID149/ChoiceGroup/SelectedChoices} was petition-able:

Not at all Petition-able	Highly Petition-able

How clear or vivid are your memories of the experience?

Completely vague or Incomprehensible	Completely Vivid

Realism

Sometimes during altered states of consciousness, the experiences can feel real on a superficial level (e.g. like imagery that occurs before sleep onset that you know is not real). However sometimes the experiences have a vivid sense of embodied presence in this world or another world that is similar in vividness to everyday normal consciousness.

Compared to normal everyday waking consciousness, please rate from 0 (slider all the way to the left) to 100 (slider completely to the right) how real the encounter with the previously identified ${q://QID149/ChoiceGroup/SelectedChoices} seemed to you.

	Not at all	Completely
Superficial dream-like level of reality		
Reality similar to everyday normal consciousness		
More real than everyday normal reality		

MEQ

Looking back on the entirety of your encounter, please rate the degree to which at any time during that encounter you experienced the following phenomena. Answer each question according to your feelings, thoughts, and experiences at the time of the encounter. In making each of your ratings, use the following scale:

0 - none; not at all
1 - so slight cannot decide
2 - slight
3 - moderate
4 - strong (equivalent in degree to any other strong experience)
5 - extreme (more than any other time in my life and stronger than 4)

MEQ - 30 ITEMS

1. Loss of your usual sense of time.

○

0 - none; not at all
1 - so slight cannot decide
2 - slight
3 - moderate
4 - strong (equivalent in degree to any previous strong experience or expectation of this description)
5 - extreme (more than ever before in my life and stonger than 4)

2. Experience of amazement.

0 - none; not at all
1 - so slight cannot decide
2 - slight
3 - moderate
4 - strong (equivalent in degree to any previous strong experience or expectation of this description)
5 - extreme (more than ever before in my life and stonger than 4)

3. Sense that the experience cannot be described adequately in words.

0 - none; not at all
1 - so slight cannot decide
2 - slight
3 - moderate
4 - strong (equivalent in degree to any previous strong experience or expectation of this description)
5 - extreme (more than ever before in my life and stonger than 4)

MEQ - 30 ITEMS

4. Gain of insightful knowledge experienced at an intuitive level.

0 - none; not at all
1 - so slight cannot decide
2 - slight

- 3 - moderate
- 4 - strong (equivalent in degree to any previous strong experience or expectation of this description)
- 5 - extreme (more than ever before in my life and stonger than 4)

5. Feeling that you experienced eternity or infinity.

- 0 - none; not at all
- 1 - so slight cannot decide
- 2 - slight
- 3 - moderate
- 4 - strong (equivalent in degree to any previous strong experience or expectation of this description)
- 5 - extreme (more than ever before in my life and stonger than 4)

6. Experience of oneness or unity with objects and/or persons perceived in your surroundings.

- 0 - none; not at all
- 1 - so slight cannot decide
- 2 - slight
- 3 - moderate
- 4 - strong (equivalent in degree to any previous strong experience or expectation of this description)
- 5 - extreme (more than ever before in my life and stonger than 4)

MEQ - 30 ITEMS

7. Loss of your usual sense of space.

- 0 - none; not at all
- 1 - so slight cannot decide
- 2 - slight
- 3 - moderate
- 4 - strong (equivalent in degree to any previous strong experience or expectation of this description)
- 5 - extreme (more than ever before in my life and stonger than 4)

8. Feelings of tenderness and gentleness.

- 0 - none; not at all
- 1 - so slight cannot decide
- 2 - slight
- 3 - moderate
- 4 - strong (equivalent in degree to any previous strong experience or expectation of this description)
- 5 - extreme (more than ever before in my life and stonger than 4)

9. Certainty of encounter with ultimate reality (in the sense of being able to "know" and "see" what is really *real*) at some time during your experience.

- 0 - none; not at all
- 1 - so slight cannot decide
- 2 - slight
- 3 - moderate
- 4 - strong (equivalent in degree to any previous strong experience or expectation of this description)
- 5 - extreme (more than ever before in my life and stonger than 4)

MEQ - 30 ITEMS

10. Feeling that you could not do justice to your experience by describing it in words.

- 0 - none; not at all
- 1 - so slight cannot decide
- 2 - slight
- 3 - moderate
- 4 - strong (equivalent in degree to any previous strong experience or expectation of this description)
- 5 - extreme (more than ever before in my life and stonger than 4)

11. Loss of usual awareness of where you were.

- 0 - none; not at all
- 1 - so slight cannot decide
- 2 - slight
- 3 - moderate
- 4 - strong (equivalent in degree to any previous strong experience or expectation of this description)
- 5 - extreme (more than ever before in my life and stonger than 4)

12. Feelings of peace and tranquility.

- 0 - none; not at all
- 1 - so slight cannot decide
- 2 - slight
- 3 - moderate
- 4 - strong (equivalent in degree to any previous strong experience or expectation of this description)
- 5 - extreme (more than ever before in my life and stonger than 4)

MEQ - 30 ITEMS

13. Sense of being "outside of" time, beyond past and future.

- 0 - none; not at all
- 1 - so slight cannot decide
- 2 - slight
- 3 - moderate
- 4 - strong (equivalent in degree to any previous strong experience or expectation of this description)
- 5 - extreme (more than ever before in my life and stonger than 4)

14. Freedom from the limitations of your personal self and feeling a unity or bond with what was felt to be greater than your personal self.

- 0 - none; not at all
- 1 - so slight cannot decide

- 2 - slight
- 3 - moderate
- 4 - strong (equivalent in degree to any previous strong experience or expectation of this description)
- 5 - extreme (more than ever before in my life and stonger than 4)

15. Sense of being at a spiritual height.

- 0 - none; not at all
- 1 - so slight cannot decide
- 2 - slight
- 3 - moderate
- 4 - strong (equivalent in degree to any previous strong experience or expectation of this description)
- 5 - extreme (more than ever before in my life and stonger than 4)

MEQ - 30 ITEMS

16. Experience of pure Being and pure awareness (beyond the world of sense impressions).

- 0 - none; not at all
- 1 - so slight cannot decide
- 2 - slight
- 3 - moderate
- 4 - strong (equivalent in degree to any previous strong experience or expectation of this description)
- 5 - extreme (more than ever before in my life and stonger than 4)

17. Experience of ecstasy.

- 0 - none; not at all
- 1 - so slight cannot decide
- 2 - slight
- 3 - moderate
- 4 - strong (equivalent in degree to any previous strong experience or expectation of this description)

- 5 - extreme (more than ever before in my life and stonger than 4)

18. Experience of the insight that "all is One."

- 0 - none; not at all
- 1 - so slight cannot decide
- 2 - slight
- 3 - moderate
- 4 - strong (equivalent in degree to any previous strong experience or expectation of this description)
- 5 - extreme (more than ever before in my life and stonger than 4)

MEQ - 30 ITEMS

19. Being in a realm with no space boundaries.

- 0 - none; not at all
- 1 - so slight cannot decide
- 2 - slight
- 3 - moderate
- 4 - strong (equivalent in degree to any previous strong experience or expectation of this description)
- 5 - extreme (more than ever before in my life and stonger than 4)

20. Experience of oneness in relation to an "inner world" within.

- 0 - none; not at all
- 1 - so slight cannot decide
- 2 - slight
- 3 - moderate
- 4 - strong (equivalent in degree to any previous strong experience or expectation of this description)
- 5 - extreme (more than ever before in my life and stonger than 4)

21. Sense of reverence.

- 0 - none; not at all
- 1 - so slight cannot decide
- 2 - slight
- 3 - moderate
- 4 - strong (equivalent in degree to any previous strong experience or expectation of this description)
- 5 - extreme (more than ever before in my life and stonger than 4)

MEQ - 30 ITEMS

22. Experience of timelessness.

- 0 - none; not at all
- 1 - so slight cannot decide
- 2 - slight
- 3 - moderate
- 4 - strong (equivalent in degree to any previous strong experience or expectation of this description)
- 5 - extreme (more than ever before in my life and stonger than 4)

23. You are convinced now, as you look back on your experience, that in it you encountered ultimate reality (i.e. that you "knew" and "saw" what was really *real).*

- 0 - none; not at all
- 1 - so slight cannot decide
- 2 - slight
- 3 - moderate
- 4 - strong (equivalent in degree to any previous strong experience or expectation of this description)
- 5 - extreme (more than ever before in my life and stonger than 4)

24. Feeling that you experienced something profoundly sacred and holy.

- 0 - none; not at all
- 1 - so slight cannot decide
- 2 - slight
- 3 - moderate
- 4 - strong (equivalent in degree to any previous strong experience or expectation of this description)
- 5 - extreme (more than ever before in my life and stonger than 4)

MEQ - 30 ITEMS

25. Awareness of the life or living presence in all things.

- 0 - none; not at all
- 1 - so slight cannot decide
- 2 - slight
- 3 - moderate
- 4 - strong (equivalent in degree to any previous strong experience or expectation of this description)
- 5 - extreme (more than ever before in my life and stonger than 4)

26. Experience of the fusion of your personal self into a larger whole.

- 0 - none; not at all
- 1 - so slight cannot decide
- 2 - slight
- 3 - moderate
- 4 - strong (equivalent in degree to any previous strong experience or expectation of this description)
- 5 - extreme (more than ever before in my life and stonger than 4)

27. Sense of awe or awesomeness.

- 0 - none; not at all
- 1 - so slight cannot decide
- 2 - slight

- 3 - moderate
- 4 - strong (equivalent in degree to any previous strong experience or expectation of this description)
- 5 - extreme (more than ever before in my life and stonger than 4)

MEQ - 30 ITEMS

28. Experience of unity with ultimate reality.

- 0 - none; not at all
- 1 - so slight cannot decide
- 2 - slight
- 3 - moderate
- 4 - strong (equivalent in degree to any previous strong experience or expectation of this description)
- 5 - extreme (more than ever before in my life and stonger than 4)

29. Feeling that it would be difficult to communicate your own experience to others who have not had similar experiences.

- 0 - none; not at all
- 1 - so slight cannot decide
- 2 - slight
- 3 - moderate
- 4 - strong (equivalent in degree to any previous strong experience or expectation of this description)
- 5 - extreme (more than ever before in my life and stonger than 4)

30. Feelings of joy.

- 0 - none; not at all
- 1 - so slight cannot decide
- 2 - slight
- 3 - moderate
- 4 - strong (equivalent in degree to any previous strong experience or expectation of this description)

5 - extreme (more than ever before in my life and stonger than 4)

Theology Background

Thank you for completing the previous section of the study answering questions about your encounter. In the following questions we will ask if there have been any persisting and long term changes that you consider due to your experience and your contemplation of that experience. Additionally, we will assess how personally impactful you feel these experiences were.

Which of the following would have best characterized your religious orientation ***immediately before your encounter*** with the ${q://QID149/ChoiceGroup/SelectedChoices} previously identified?

Agnostic	Falun Gong	Native American	Secular Humanist
Atheist	Hindu	Nature-based	Shinto
Baha'i	Islam	Pagan	Taosim
Buddhist	Jainism	Pantheist (belief in the divinity of the universe)	Unitarian Universalist
Christian	Jewish	Scientology	Zoroastrian
Earth-based	Nation of Islam	Secular (no religious or spiritual orientation)	Other

Please describe briefly (200 characters or less):

Please specify:

Catholic	Methodist	Calvanist
Eastern Orthodox	Quaker	Adventist
Episcopalian	Pentecostal	Congregationalist

○	○	○
○ Baptist	○ Nondenomenational	○ Mormon
○ Lutheran	○ Anglican	○ Other
○ Presbyterian		

Please specify:

- Sunni
- Shi'ah
- Khawarij
- Sufi
- Other

Please specify:

- Orthodox
- Conservative
- Reform
- Reconstructionist
- Other

Did your religious orientation change in response to your encounter and your contemplations of the encounter with the ${q://QID149/ChoiceGroup/SelectedChoices} previously identified?

Yes	No
○	○

Which of the following would best characterize your religious orientation ***after your encounter*** with the ${q://QID149/ChoiceGroup/SelectedChoices} previously identified?

○ Agnostic	○ Falun Gong	○ Native American	○ Secular Humanist
○ Atheist	○ Hindu	○ Nature-based	○ Shinto
○ Baha'i	○ Islam	○ Pagan	○ Taosim

- Buddhist
- Jainism
- Pantheist (belief in the divinity of the universe)
- Unitarian Universalist
- Christian
- Jewish
- Scientology
- Zoroastrian
- Earth-based
- Nation of Islam
- Secular (no religious or spiritual orientation)
- Other

Please describe briefly (200 characters or less):

Please specify:

- Catholic
- Methodist
- Calvanist
- Eastern Orthodox
- Quaker
- Adventist
- Episcopalian
- Pentecostal
- Congregationalist
- Baptist
- Nondenomenational
- Mormon
- Lutheran
- Anglican
- Other
- Presbyterian

Please specify:

- Sunni
- Shi'ah
- Khawarij
- Sufi
- Other

Please specify:

- Orthodox
- Conservative
- Reform
- Reconstructionist
- Other

How would you best describe this change in religious orientation?

- +3. Strong positive change that I consider desirable
- +2. Moderate positive change that I consider desirable
- +1. Slight positive change that I consider desirable
- 0. No change
- -1. Slight negative change that I consider detrimental
- -2. Moderate negative change that I consider detrimental
- -3. Strong negative change that I consider detrimental

Do you believe that the experience and your contemplation of that experience has led to longterm and persisting changes in your **personal contemplative, prayer, or meditation practices**?

- +3. Increased strongly
- +2. Increased moderately
- +1. Increased slightly
- 0. No change
- -1. Decreased slightly
- -2. Decreased moderately
- -3. Decreased strongly

Do you consider that change in your **personal contemplative, prayer, or meditation practices** to be:

Desirable	Undesirable
○	○

Do you believe that the experience and your contemplation of that experience has led to longterm and persisting changes in your **appreciation or understanding of religious or spiritual traditions other than your own**?

- +3. Increased strongly
- +2. Increased moderately
- +1. Increased slightly
- 0. No change
- -1. Decreased slightly
- -2. Decreased moderately
- -3. Decreased strongly

Do you consider that change in your **appreciation or understanding of religious or spiritual traditions other than your own** to be:

Desirable	Undesirable
○	○

Did the experience change your attitudes about death?

- +3. Strong positive change that I consider desirable
- +2. Moderate positive change that I consider desirable
- +1. Slight positive change that I consider desirable
- 0. No change
- -1. Slight negative change that I consider detrimental
- -2. Moderate negative change that I consider detrimental
- -3. Strong negative change that I consider detrimental

Did the experience change your curiosity or interest in death?

- +3. Strong positive change that I consider desirable
- +2. Moderate positive change that I consider desirable
- +1. Slight positive change that I consider desirable
- 0. No change

- -1. Slight negative change that I consider detrimental
- -2. Moderate negative change that I consider detrimental
- -3. Strong negative change that I consider detrimental

Did the experience change your positive attitudes about your own death?

- +3. Strong positive change that I consider desirable
- +2. Moderate positive change that I consider desirable
- +1. Slight positive change that I consider desirable
- 0. No change
- -1. Slight negative change that I consider detrimental
- -2. Moderate negative change that I consider detrimental
- -3. Strong negative change that I consider detrimental

Did the experience change your fear of death?

Decrease	No Change	Increase
◯	◯	◯

Thinking back on the experience and your contemplation of that experience, please answer the following questions:

How **personally meaningful** was your experience and your contemplation of that experience?

- 1. No more than routine, everyday experiences
- 2. Similar to meaningful experiences that occur on average once or more a week
- 3. Similar to meaningful experiences that occur on average once a month
- 4. Similar to meaningful experiences that occur on average once a year
- 5. Similar to meaningful experiences that occur on average once every 5 years
- 6. Among the 10 most meaningful experiences of my life
- 7. Among the 5 most meaningful experiences of my life
- 8. The single most meaningful experience of my life

How **spiritually significant** was your experience and your contemplation of that experience?

- 1. No more than routine, everyday experiences
- 2. Similar to spiritually significant experiences that occur on average once or more a week
- 3. Similar to spiritually significant experiences that occur on average once a month
- 4. Similar to spiritually significant experiences that occur on average once a year
- 5. Similar to spiritually significant experiences that occur on average once every 5 years
- 6. Among the 10 most spiritually significant experiences of my life
- 7. Among the 5 most spiritually significant experiences of my life
- 8. The single most spiritually significant experience of my life

How **psychologically challenging** was the most psychologically challenging portions of the experience?

- 1. No more than routine, everyday experiences
- 2. Similar to difficult or challenging experiences that occur on average once or more a week
- 3. Similar to difficult or challenging experiences that occur on average once a month
- 4. Similar to difficult or challenging experiences that occur on average once a year
- 5. Similar to difficult or challenging experiences that occur on average once every 5 years
- 6. Among the 10 most difficult or challenging experiences of my life
- 7. Among the 5 most difficult or challenging experiences of my life
- 8. The single most difficult or challenging experience of my life

How **personally psychologically insightful** to you was the experience and your contemplation of that experience?

[Note: Some people report experiences of significant personal psychological insight based on their contemplation of their experiences. Personal psychological insight refers to realizations about personality, relationships, behavioral patterns, or emotions. Personal psychological insight can occur in complete absence of a spiritual/mystical insight, although spiritual mystical insight can sometimes prompt personal psychological insight.]

- 1. No more than routine, everyday psychologically insightful experiences
- 2. Similar to psychologically insightful experiences that occur on average once or more a week
- 3. Similar to psychologically insightful experiences that occur on average once a month
- 4. Similar to psychologically insightful experiences that occur on average once a year
- 5. Similar to psychologically insightful experiences that occur on average once every 5 years
- 6. Among the 10 most psychologically insightful experiences of my life

- 7. Among the 5 most psychologically insightful experiences of my life
- 8. The single most psychologically insightful experiences of my life

Did the experience occur while you were alone or in the presence of others?

I was alone	I was in the presence of others
◯	◯

Around how many people were you in the presence of?

Did you feel any social support during the experience?

Yes	No
◯	◯

How important do you think social support was to the experience?

- 0 - not at all
- 1 - so slight, cannot decide
- 2 - slight
- 3 - moderate
- 4 - strong
- 5 - extreme

Do you believe that the experience and your contemplation of that experience has led to longterm and persisting changes in your current sense of **personal well-being** or **life satisfaction**?

- +3. Strong positive change that I consider desirable
- +2. Moderate positive change that I consider desirable
- +1. Slight positive change that I consider desirable

0. No change

-1. Slight negative change that I consider undesirable

-2. Moderate negative change that I consider undesirable

-3 Strong negative change that I consider undesirable

Do you believe that the experience and your contemplation of that experience has led to longterm and persisting changes in the sense of **your life's purpose**?

+3. Strong positive change that I consider desirable

+2. Moderate positive change that I consider desirable

+1. Slight positive change that I consider desirable

0. No change

-1. Slight negative change that I consider undesirable

-2. Moderate negative change that I consider undesirable

-3 Strong negative change that I consider undesirable

Do you believe that the experience and your contemplation of that experience has led to longterm and persisting changes in the sense of **your life's meaning**?

+3. Strong positive change that I consider desirable

+2. Moderate positive change that I consider desirable

+1. Slight positive change that I consider desirable

0. No change

-1. Slight negative change that I consider undesirable

-2. Moderate negative change that I consider undesirable

-3 Strong negative change that I consider undesirable

Do you believe that the experience and your contemplation of that experience has led to longterm and persisting changes in your **social relationships as a whole** (e.g., relationships with family members, friends, neighbors, co-workers, strangers, etc.)?

+3. Strong positive change that I consider desirable

+2. Moderate positive change that I consider desirable

+1. Slight positive change that I consider desirable

0. No change

-1. Slight negative change that I consider undesirable

-2. Moderate negative change that I consider undesirable

-3 Strong negative change that I consider undesirable

Do you believe that the experience and your contemplation of that experience has led to longterm and persisting changes in your **spiritual awareness in everyday life**?

+3. Strong positive change that I consider desirable

+2. Moderate positive change that I consider desirable

+1. Slight positive change that I consider desirable

0. No change

-1. Slight negative change that I consider undesirable

-2. Moderate negative change that I consider undesirable

-3 Strong negative change that I consider undesirable

Do you believe that the experience and your contemplation of that experience has led to longterm and persisting changes in your **attitudes about life**?

+3. Strong positive change that I consider desirable

+2. Moderate positive change that I consider desirable

+1. Slight positive change that I consider desirable

0. No change

-1. Slight negative change that I consider undesirable

-2. Moderate negative change that I consider undesirable

-3 Strong negative change that I consider undesirable

Do you believe that the experience and your contemplation of that experience has led to longterm and persisting changes in your **attitudes about self**?

+3. Strong positive change that I consider desirable

+2. Moderate positive change that I consider desirable

+1. Slight positive change that I consider desirable

0. No change

- -1. Slight negative change that I consider undesirable
- -2. Moderate negative change that I consider undesirable
- -3 Strong negative change that I consider undesirable

Do you believe that the experience and your contemplation of that experience has led to longterm and persisting changes in your **mood**?

- +3. Strong positive change that I consider desirable
- +2. Moderate positive change that I consider desirable
- +1. Slight positive change that I consider desirable
- 0. No change
- -1. Slight negative change that I consider undesirable
- -2. Moderate negative change that I consider undesirable
- -3 Strong negative change that I consider undesirable

Do you believe that the experience and your contemplation of that experience has led to longterm and persisting **behavioral changes?**

- +3. Strong positive change that I consider desirable
- +2. Moderate positive change that I consider desirable
- +1. Slight positive change that I consider desirable
- 0. No change
- -1. Slight negative change that I consider undesirable
- -2. Moderate negative change that I consider undesirable
- -3 Strong negative change that I consider undesirable

Do you believe that the experience and your contemplation of that experience has led to longterm and persisting changes in how **spiritual** you are?

- +3. Strong positive change that I consider desirable
- +2. Moderate positive change that I consider desirable
- +1. Slight positive change that I consider desirable
- 0. No change
- -1. Slight negative change that I consider undesirable

-2. Moderate negative change that I consider undesirable

-3 Strong negative change that I consider undesirable

INSPIRIT + Other

Some people's experience of continuing encounters with ${q://QID149/ChoiceGroup/SelectedChoices} are limited only to occasions after they have taken a classic hallucinogen, while other people have experiences that occur in the absence of taking a classic hallucinogen. You have completed this survey on the basis of your most memorable experience after taking a classic hallucinogen. Although possibly not as memorable, after the experience you described, have you ever had such an experience in the absence of taking classic hallucinogens?

Yes	No
○	○

Such continuing encounters can vary in measures of intensity. Considering all such experiences of **any intensity (weak, moderate, strong)**, what is the frequency of such continuing encounters with the previously identified ${q://QID149/ChoiceGroup/SelectedChoices} in the **absence of taking classic hallucinogens**?

- more than twice a day
- twice a day
- once a day
- 5-6x/week
- 3-4x/week
- 1-2x/week
- 1-3x/month
- less than 1x/month

Such continuing encounters can vary in measures of intensity. Considering all such experiences of **moderate or strong intensity**, what is the frequency of such continuing encounters with the previously identified ${q://QID149/ChoiceGroup/SelectedChoices} in the **absence of taking classic hallucinogens**?

- more than twice a day
- twice a day
- once a day
- 5-6x/week
- 3-4x/week
- 1-2x/week

- 1-3x/month
- less than 1x/month

Final set of questions about your general spiritual or religious beliefs and experience:

Thinking very broadly, do you believe there is some form of continuance after death?

Yes	Possibly	No, absolutely not
○	○	○

What do you believe happens after you die?

Note: Check all that could apply

- ☐ Nothing, I will cease to exist
- ☐ I will go to a positive realm (e.g., heaven, nirvana, paradise, etc.)
- ☐ I will go to a negative realm (e.g., hell, a realm of suffering, etc.)
- ☐ I will go to a positive realm (e.g., heaven, nirvana, paradise, etc.) or a negative realm (e.g., hell, a realm of suffering, etc.)
- ☐ I will go to a neutral realm, separate from the conventional positive and negative realms
- ☐ I will be reincarnated
- ☐ There is a continuity of some form of consciousness
- ☐ There is a continuity of spirit
- ☐ There is a continuity of energy
- ☐ There is a continuity of soul
- ☐ There is a continuity through nature
- ☐ There is continuity through family and friends
- ☐ Other

Please explain briefly (250 characters max):

Do your beliefs about what happens when you die bring you:

Comfort	Discomfort	Neither comfort nor discomfort	Both comfort and discomfort
○	○	○	○

Some believe that there are only evil acts (e.g., unjustified killing of an innocent), however, others believe that there are people who are truly evil (e.g., agents of the devil).

Do you think that there are people who are truly evil, or only people committing evil acts out of circumstance (e.g., ignorance, mental illness, or socialized to commit evil acts)?

Yes, there are people who are truly evil (e.g., agents of the devil)	No, there are only people committing evil acts out of ignorance	I do not know
○	○	○

Do you believe in the literal interpretation of the seminal religious text that forms the foundation of your religious understanding (i.e. the events in that text occurred exactly as they are described)?

Yes	No
○	○

The following questions concern your spiritual or religious beliefs and experiences. There are no right or wrong answers. For each question, select the response that is most true for you.

1. How strongly religious (or spiritually oriented) do you consider yourself to be?

- ○ Not at all
- ○ Not very strong
- ○ Somewhat strong
- ○

Strong

2. About how often do you spend time on religious or spiritual practices?

- Several times per day - several times per week
- Once per week - several times per month
- Once per month - several times per year
- Once a year or less

3. How often have you felt as though you were very close to a powerful spiritual force that seemed to lift you outside yourself?

- Never
- Once or twice
- Several times
- Often

People have many different definitions of the "Higher Power" that we often call "God." <u>Please use your definition of God when answering the following questions:</u>

4. How close do you feel to God?

- I don't believe in God
- Not very close
- Somewhat close
- Extremely close

5. Have you ever had an experience that has convinced you that God exists?

- Yes
- No

6. Indicate whether you agree or disagree with this statement: "God dwells within you."

- Definitely disagree
- Tend to disagree
- Tend to agree
- Definitely agree

The following list describes spiritual experiences that some people have had. Please indicate if you have had any of these experiences and the extent to which each of them has affected your belief in God.

	I have never had this experience	I have had this experience and it:		
		Did not strengthen belief in God	Strengthened belief in God	Convinced me of God's existence
A. An experience of God's energy or presence	○	○	○	○
B. An experience of a great spiritual figure (e.g., Jesus, Mary, Elijah, Buddah)	○	○	○	○
C. An experience of angels or guiding spirits	○	○	○	○
D. An experience of communication with someone who has died	○	○	○	○
E. Meeting or listening to a spiritual teacher or master	○	○	○	○

	I have never had this experience	I have had this experience and it:		
		Did not strengthen belief in God	Strengthened belief in God	Convinced me of God's existence
F. An overwhelming experience of love	○	○	○	○
G. An experience of profound inner peace	○	○	○	○
H. an experience of complete joy and ectsasy	○	○	○	○
I. A miraculous (or not normally	○	○	○	○

occurring) event				
J. A healing of your body or mind (or witnessed such a healing)	○	○	○	○

	**I have never had this experience**	**I have had this experience and it:** Did not strengthen belief in God	Strengthened belief in God	Convinced me of God's existence
K. A feeling of unity with the earth and all living beings	○	○	○	○
L. An experience with near death or life after death	○	○	○	○
M. Other	○	○	○	○

Which of the following statements do you most closely identify with?

I have no doubts that a God or Gods do **not** exist	I do **not** believe that a God or Gods exist	I am not sure	I believe a God or Gods exist	I have no doubts that a God or Gods exist
○	○	○	○	○

You have previously identified that you most closely identify with the statement, "${q://QID441/ChoiceGroup/SelectedChoices}"

Please rate the degree of certainty with which you believe your previous answer:

Completely Uncertain — Completely Certain

0 — 100

In the previous question you indicated that you were not sure whether or not a God or Gods exist. Using the below, please indicate where you fall on the continuum of belief:

I do not believe a God or Gods exist — I do believe a God or Gods exist

0 100

Final Thoughts

Optional question:

This text box is a final opportunity for you to provide any additional information or insights you might wish to share about your encounter with the previously identified ${q://QID149/ChoiceGroup/SelectedChoices}.

Although we have already asked you many specific questions, we recognize that there still may be important details of your story that you would like us to know. The space below is an opportunity for you to tell us about these other details.

If you encountered any technical difficulties during completion of this survey we would appreciate it if you would briefly describe them briefly in the text box below:

The accuracy of the responses we collect and analyze in this anonymous survey is very important to us. If you think you did not provide reasonably accurate information when completing this survey (e.g. you became distracted and

lost focus), we would appreciate you letting us know so that the data can be excluded from analysis.

- Please discard my responses
- Please keep my responses because I believe them to be reasonably accurate
